# Specificity-driven cell-gene graph learning identifies rare cell states in single-cell and spatial transcriptomic data

**DOI:** 10.1007/s44307-026-00121-y

**Published:** 2026-07-16

**Authors:** Jinjin Huang, Xuanzhe Xia, Feng Luo, Lianghu Qu, Xiao Feng, Lingling Zheng

**Affiliations:** 1 School of Agriculture and Biotechnology, Sun Yat-Sen University Shenzhen Campus, Shenzhen, 518107 China; 2 MOE Key Laboratory of Gene Function and Regulation, State Key Laboratory for Biocontrol, Guangdong Provincial Key Laboratory of Plant Stress, Innovation Center for Evolutionary Synthetic Biology, School of Life Sciences, Sun Yat-Sen University, Guangzhou, 510275 China

**Keywords:** Rare cell identification, Single-cell RNA sequencing, Heterogeneous graph transformer, Cell-gene graph learning, Spatial transcriptomics

## Abstract

Detecting rare cell populations that drive development, differentiation, and disease-associated transformation remains a central challenge in biology and medicine. Although these populations often represent promising targets for intervention, they are difficult to resolve from single-cell transcriptomic data because most methods rely on homophily-based cell–cell similarity, which can merge rare cells into dominant populations and mask their subtle transcriptional signatures. The challenge is further amplified in multi-sample analyses, where batch correction can dilute rare-cell-specific signals. Here, we present scFormer, a heterogeneous graph transformer (HGT) framework for sensitive and robust rare-cell discovery. scFormer constructs a Z-score-guided cell-gene heterogeneous graph in which highly specific marker genes serve as informational bridges, embedding rare-cell features directly into the graph topology rather than inferring them from global neighbors. This design provides a clear biological rationale for rare-cell recovery, as low-abundance cells can remain connected through shared high-specificity genes even when local cell–cell neighborhoods are sparse. An integrated optimization strategy jointly performs representation learning, clustering, and optional batch correction, enabling rare-cell discovery while preserving biological structure. Across 125 simulated and 18 real datasets, scFormer consistently achieved competitive or superior performance relative to existing approaches. Applied to diverse multi-sample single-cell and spatial transcriptomics datasets, scFormer recovered known but weakly represented populations and revealed previously obscured cell states, including proliferative club cells in the airway epithelium, revival stem cells during intestinal regeneration, and rare embryonic cell states from spatial transcriptomics. Overall, scFormer provides a unified framework for identifying biologically meaningful rare populations while mitigating batch effects in multi-sample datasets.

**Supplementary Information:**

The online version contains supplementary material available at 10.1007/s44307-026-00121-y.

## Introduction

Multicellular tissues are composed of a spectrum of cell types and dynamic states, including low-frequency populations that are often transient, spatially restricted, or activated only under stress or injury. These rare populations are of particular translational interest in oncology: even when they represent only a small fraction of cells (< 1%–5%), they can disproportionately influence tumor progression, immune evasion, therapeutic resistance, and relapse (Chu et al. 2024; Galassi et al. 2024; Goddard et al. 2024; Shaffer et al. 2017). Beyond cancer, rare populations also mark lineage commitment, regenerative trajectories, and specialized functional niches across normal and diseased tissues (Belarif et al. 2018; Ross et al. 1993; Wijnands et al. 2023). Resolving these populations is therefore central not only to understanding tissue development and homeostasis, but also to improving disease stratification and precision therapeutic intervention.

Single-cell RNA sequencing (scRNA-seq) now enables comprehensive profiling of cellular heterogeneity at unprecedented resolution (Lu et al. 2024; Liao et al. 2025; Potter 2018; Choi and Kim 2019; Jaitin et al. 2014; Hu et al. 2024; Sang and Kong 2024). However, rare-cell identification faces two key technical challenges. First, transcriptional signals from rare cells are easily masked by abundant cell types, limiting feature extraction (Zhou et al. 2024). Second, batch effects arising from differences in experimental protocols, sequencing depth, and sample handling introduce systematic variation that can confound downstream analyses, especially in multi-sample studies (Argelaguet et al. 2021).

Numerous computational strategies have been proposed to address rare-cell detection, yet fundamental limitations persist. Rarity-based approaches (for example, FiRE (Jindal et al. 2018) and GapClust (Fa et al. 2021)) and feature-selection strategies such as GiniClust3 (Dong and Yuan 2020) quantify cellular uniqueness but remain sensitive to noise and subtle expression shifts. Clustering-based methods (RaceID3 (Herman et al. 2018), CellSIUS (Wegmann et al. 2019), scCAD (Xu et al. 2024)) and feature-learning approaches such as SCMER (Liang et al. 2021) depend primarily on global cell–cell similarity. As a consequence, when rare cells lack sufficient neighbors to form distinct clusters, their signatures are often over-smoothed and absorbed into dominant lineages.

A critical and largely unresolved issue concerns the interaction between rare-cell detection and batch correction. Dedicated integration tools such as Harmony (Korsunsky et al. 2019), BBKNN (Polański et al. 2020), ComBat (Zhang et al. 2020) and MNN Correct (Haghverdi et al. 2018) are optimized to align global distributions across samples, not to preserve rare signals. Over-correction may inadvertently merge true rare populations into transcriptionally similar abundant types. Conversely, applying rare-cell detection without integration risks inflating false positives driven by technical variation rather than biology (Yong et al. 2021). This tension motivates the need for methods that remove batch effects while preserving weak but biologically meaningful signals.

Graph neural network and transformer-based models have recently expanded the methodological toolkit for single-cell analysis. For example, scMGCA uses a graph-embedding autoencoder to learn cell–cell topology and cluster assignments across single-cell platforms (Yu et al. 2023), whereas scHeteroNet introduces a heterophily-aware graph neural network for cell-type annotation and novel-cell detection (Liu et al. 2025). Beyond cell–cell graph modeling, heterogeneous graph transformer (HGT)-based frameworks have shown that distinct biological entities and relation types can be modeled explicitly in single-cell data. DeepMAPS represents cells and genes as distinct node types and uses HGT-based message passing for single-cell biological network inference (Ma et al. 2023), while MarsGT applies graph transformer learning to rare-population inference from single-cell multi-omics data (Wang et al. 2024). These studies demonstrate the value of GNNs, graph transformers, and cell-gene heterogeneous graphs in single-cell analysis. However, existing graph-based frameworks still leave an important challenge for rare-cell discovery. Rare populations are often too sparse to form robust cell–cell neighborhoods, and batch correction may further dilute weak but biologically meaningful variation. A method designed for this setting should therefore not only represent cells and genes in a heterogeneous graph, but also construct a rare-cell-oriented cell-gene topology and learning objective that preserve cell-specific transcriptional signals before they are absorbed into abundant populations.

Here, we present scFormer, a unified computational framework that uses HGTs for rare-cell identification with optional batch effect correction. Rather than relying on conventional cell–cell similarity networks that can obscure rare signals, scFormer converts cell-specific marker evidence into the topology of a cell-gene heterogeneous graph. Each cell is connected to a compact set of highly informative genes, allowing rare transcriptional programs to be represented by direct cell-gene edges before graph propagation. In this topology, highly specific marker genes serve as informational bridges, encoding biological priors directly into graph structure. This design is intended to preserve minority transcriptional programs that are often smoothed away in homophily-driven cell–cell graphs. The HGT then propagates information through node- and relation-aware attention, enabling cell and gene nodes to exchange information while retaining their distinct biological roles. scFormer further integrates representation learning, clustering regularization and optional batch correction within a unified objective, allowing rare-cell discovery across both single-sample and multi-sample settings. We validated scFormer on 125 simulated and 18 real scRNA-seq datasets spanning diverse tissues and biological contexts, and observed improved benchmark performance and robustness compared with existing methods. Beyond recapitulating known rare cell types, scFormer identified previously uncharacterized rare populations in airway epithelium and intestinal tissue, and showed applicability to spatial transcriptomics by identifying rare embryonic cell states that could be examined within their original tissue coordinates. Together, these results establish scFormer as a practical framework for uncovering functionally important rare cell populations, with broad implications for developmental biology, immunology, and precision medicine.

## Methods

### Data preprocessing

Single-cell RNA-seq and spatial transcriptomic datasets were represented as a non-negative expression matrix $\mathbf{C}\in {\mathbb{R}}_{\ge 0}^{N\times G}$, where $N$ is the number of cells or spatial profiles and $G$ is the number of detected genes. Each row corresponds to a cell or spatial profile, and each column corresponds to a gene. For raw count matrices, library-size normalization and log transformation were first applied:

1 $$\begin{array}{c}{\mathbf{X}}_{ij}=\mathrm{log}\left(1+\frac{{10}^{4}{C}_{ij}}{{\sum}_{g=1}^{G}{C}_{ig}}\right),\end{array}$$

where ${\mathbf{C}}_{ij}$ denotes the count of gene $j$ in cell or spatial profile $i$, and $\mathbf{X}$ denotes the resulting non-negative normalized expression matrix. For datasets distributed as preprocessed non-negative expression matrices, the provided expression values were used directly as $\mathbf{X}$, followed by the same gene-wise standardization procedure to obtain $\mathbf{Z}$. This preprocessing workflow was applied to both scRNA-seq and spatial transcriptomic expression matrices.

To quantify cell-specific gene enrichment, gene-wise standardization was then performed on $\mathbf{X}$. For each gene $j$, the mean expression ${\mu}_{j}$ and standard deviation ${\sigma}_{j}$ were computed across all cells or spatial profiles, and the standardized expression matrix $\mathbf{Z}$ was defined as:

2 $$\begin{array}{c}{\mathbf{Z}}_{ij}=\frac{{\mathbf{X}}_{ij}-{\mu}_{j}}{{\sigma}_{j}},\end{array}$$

Thus, $\mathbf{X}$ and $\mathbf{Z}$ serve distinct roles in scFormer. The standardized matrix $\mathbf{Z}$ was used as the specificity score matrix for cell-gene edge construction and as the source of node input features. For a cell node $i$, the input feature was its standardized expression vector ${\mathbf{Z}}_{i,:}\in {\mathbb{R}}^{G}$. For a gene node $j$, the input feature was its standardized expression profile across all cells or spatial profiles, ${\mathbf{Z}}_{:,j}\in {\mathbb{R}}^{N}$. Cell and gene features were projected to the hidden dimension by node-type-specific input encoders before HGT message passing. The non-negative matrix $\mathbf{X}$, rather than the standardized matrix $\mathbf{Z}$, was retained as the expression target for the reconstruction objective during training.

### Construction of cell-gene heterogeneous graph

#### Selecting high expression genes and forming the adjacency matrix

After obtaining the standardized expression matrix $\mathbf{Z}\in {\mathbb{R}}^{N\times G}$, scFormer constructed a cell-gene heterogeneous graph by connecting each cell to genes with high cell-specific Z-scores. Unlike traditional GNNs that rely on KNN graphs to model cell–cell relationships, which often assume homophily and may overlook heterogeneous relationships, scFormer uses genes as an intermediary bridge to link cells. This gene-mediated approach captures cell-gene associations directly and helps retain cell-specific transcriptional signals.

For each cell $i$, genes are ranked according to their standardized expression values ${\mathbf{Z}}_{ij}$. The selected gene set for cell $i$ is defined as

3 $$\begin{array}{c}{\Gamma}_{i}^{K}={\mathrm{T}\mathrm{o}\mathrm{p}\mathrm{K}}_{j}\left({\mathbf{Z}}_{ij}\right)\end{array}$$

where $K$ denotes the number of genes retained for each cell. In all main analyses, *K* = 20 was used as a fixed default graph-construction parameter rather than being optimized separately for individual datasets or selected according to evaluation labels. This value was chosen to maintain a sparse but sufficiently informative cell-gene graph. To assess whether this default choice introduced parameter-specific bias, we performed a sensitivity analysis across *K* = 5, 10, 20, 30 and 50, using 25 simulated datasets spanning different proportions of differentially expressed genes, thereby covering sparse-to-denser marker-profile settings. The results showed that rare-cell detection performance increased from smaller $K$ values to *K* = 20 and then approached a plateau, whereas larger $K$ values produced only marginal F1-score gains while increasing runtime and graph size (Supplementary Fig. 1). Here, $K$ controls the sparsity of the cell-gene graph by limiting the number of selected genes per cell.

We then construct a binary adjacency matrix $\mathbf{A}\in {\{\mathrm{0,1}\}}^{N\times G}$ as follows:

4 $$\begin{array}{c}{\mathbf A}_{ij}=\left\{\begin{array}{c}1,\;\;if\;j\in\Gamma_i^K\\0,\;otherwise\end{array},\right.\end{array}$$

Thus, ${\mathbf{A}}_{ij}=1$ indicates that gene $j$ is selected as one of the top $K$ Z-score-ranked genes for cell $i$, whereas ${\mathbf{A}}_{ij}=0$ indicates no selected cell-gene edge.

#### Defining node types

To enable heterogeneous graph modeling, we distinguish between two node types: cell nodes (indexed by $\left\{0\right.,1,...,\left.N-1\right\}$) and gene nodes (indexed by $\left\{N\right.,N+1,...,\left.N+G-1\right\}$). This indexing convention ensures that the numerical ranges of cell nodes and gene nodes do not overlap, facilitating the definition of directed edges and their types.

#### Constructing the cell-gene edge indices

We extract nonzero entries from the adjacency matrix $\mathbf{A}$ to form the selected cell-gene associations. Let

5 $$\begin{array}{c}\Omega \in \left\{\left.(i,j)|{\mathbf{A}}_{ij}=1, 0\le i<N, 0\le j<G\right\}\right.,\end{array}$$

where $\Omega$ denotes the set of all selected cell-gene pairs. Because cell and gene nodes are placed on disjoint index sets, we define a constant offset $\Delta =N$. A gene index *j*, where $0\le j<G$, is mapped to the graph node index $\Delta +j$ to distinguish it from the cell indices $\{0,...,N-1\}$.

To represent each selected association bidirectionally, we add both a gene-to-cell edge and a cell-to-gene edge. For each $(i,j)\in\Omega$, the gene-to-cell edge has source node $\Delta +j$ and target node *i*, Formally,

6 $$\begin{array}{c}{\mathcal{E}}_{\mathrm{g}\mathrm{e}\mathrm{n}\mathrm{e}\to \mathrm{c}\mathrm{e}\mathrm{l}\mathrm{l}}=\left\{\left.(\Delta +j,i)|(i,j)\in\Omega \right\}\right..\end{array}$$

For the same selected pair $(i,j)\in\Omega$, we also add the reverse cell-to-gene edge, with source node *i* and target node $\Delta +j$:

7 $$\begin{array}{c}{\mathcal{E}}_{\mathrm{c}\mathrm{e}\mathrm{l}\mathrm{l}\to \mathrm{g}\mathrm{e}\mathrm{n}\mathrm{e}}=\left\{\left.(i,\Delta +j)|(i,j)\in\Omega \right\}\right..\end{array}$$

By merging these two directed edge sets, we obtain the complete set of edges:

8 $$\begin{array}{c}E={\mathcal{E}}_{\mathrm{g}\mathrm{e}\mathrm{n}\mathrm{e}\to \mathrm{c}\mathrm{e}\mathrm{l}\mathrm{l}}\cup {\mathcal{E}}_{\mathrm{c}\mathrm{e}\mathrm{l}\mathrm{l}\to \mathrm{g}\mathrm{e}\mathrm{n}\mathrm{e}}.\end{array}$$

In practice, these edges are often stored in a $2\times \left|\left.\mathcal{E}\right|\right.$ array, where the first row contains source-node indices and the second row contains target-node indices.

Specifying edge and node types.

We also define an edge-type vector $\mathbf{edge}\_\mathbf{type}$ to annotate whether an edge represents gene to cell or cell to gene, such that:

9 $$\begin{array}{c}\mathbf{edge}\_\mathbf{type}=\left[\underbrace{\mathrm{0,0},\dots,0}_{\,\left|{\mathcal E}_{\mathrm{g}\mathrm{e}\mathrm{n}\mathrm{e}\rightarrow\mathrm{c}\mathrm{e}\mathrm{l}\mathrm{l}}\right|edges},\underbrace{\mathrm{1,1},\dots,1}_{\left|{\mathcal E}_{\mathrm{c}\mathrm{e}\mathrm{l}\mathrm{l}\rightarrow gene}\right|edges}\right].\end{array}$$

Similarly, a node-type vector $\mathbf{n}\mathbf{o}\mathbf{d}\mathbf{e}\_\mathbf{t}\mathbf{y}\mathbf{p}\mathbf{e}$ can label each node as either cell or gene:

10 $$\begin{array}{c}\mathbf{node}\_\mathbf{type}=\left[\underset{N\text{ cell nodes}}{\underbrace{\mathrm{0,0},\dots ,0}},\underset{G \, gene nodes}{\underbrace{\mathrm{1,1},\dots ,1}}\right].\end{array}$$

These annotations enable heterogeneous graph algorithms to handle different node and edge types explicitly.

### Subsampling of a cell-gene heterogeneous graph

For mini-batch training, scFormer sampled cells first and then constructed the corresponding batch-specific gene set. Before mini-batch construction, Python, NumPy, and PyTorch random seeds were fixed, and cell indices were randomly shuffled before being split into mini-batches. In the main experiments, the batch size was set to 300 cells.

For each mini-batch $B$, the gene nodes were defined as the union of the top $K$ Z-score-selected genes from all cells in that batch:

11 $${\Gamma}_{B}=\bigcup\limits_{i\in B}{\Gamma}_{i}^{K}.$$

Thus, for a mini-batch containing $\left|B\right|$ cells, each sampled cell contributes up to $K$ selected genes, and duplicated genes selected by multiple cells are merged. The number of gene nodes in a mini-batch is therefore determined by $\left|{\Gamma}_{B}\right|$, with an upper bound of $\left|B\right|\times K$, rather than by an independently fixed cell-to-gene sampling ratio.

The mini-batch subgraph was induced by the sampled cells $B$, the selected gene set ${\Gamma}_{B}$, and all corresponding bidirectional cell-gene edges between them. The only graph expansion after cell sampling was the one-hop inclusion of selected gene nodes defined by ${\Gamma}_{i}^{K}$; no additional multi-hop neighbor expansion or independent gene-node sampling was performed. Instead, the batch graph was defined directly by the specificity-based cell-gene associations established during graph construction. The cell-node features in this mini-batch were ${\mathbf{\rm Z}}_{B},:$, and the gene-node features were ${{\boldsymbol{Z}}}_{{:,\Gamma }_{B}}^{T}$, namely the standardized profiles of the selected genes across all cells or spatial profiles.

This batching strategy preserves the selected cell-gene connections within each batch rather than sampling cell and gene nodes independently. Because each sampled cell contributes its own top $K$ genes, sampled cell nodes retain their specificity-based cell-gene edges when at least one gene is retained for that cell. Gene nodes are included only if they are selected by at least one sampled cell, so isolated gene nodes are not introduced during mini-batch construction. The generated mini-batch indices were saved and reused during training, further ensuring reproducible subgraph construction across runs using the same random seed.

### Construction of the heterogeneous graph convolution network

An effective strategy for capturing the distinct characteristics of cells ($\mathbf{n}\mathbf{o}\mathbf{d}\mathbf{e}\_\mathbf{t}\mathbf{y}\mathbf{p}\mathbf{e}=0$) and genes ($\mathbf{n}\mathbf{o}\mathbf{d}\mathbf{e}\_\mathbf{t}\mathbf{y}\mathbf{p}\mathbf{e}=1$) is the application of a HGT layer. In scFormer, cell nodes and gene nodes are first projected into a common hidden dimension by node-type-specific input encoders before HGT message passing. Let ${h}_{v}^{\left(l-1\right)}\in {\mathbb{R}}^{{d}_{h}}$ denote the hidden representation of node $v$ at layer $l-1$, where ${d}_{h}$ is the hidden dimension. The number of attention heads is denoted by $H$, and the dimension of each head is ${d}_{k}={d}_{h}/H$.

For each node type $\tau \in \left\{\left.\mathrm{0,1}\right\}\right.$, where $\tau =0$ denotes cell nodes and $\tau =1$ denotes gene nodes, scFormer uses node-type-specific linear projections to compute queries, keys, and values:

12 $$\begin{aligned}&{\mathbf{Q}}^{\left(\tau \right)}={\mathbf{H}}_{\tau }^{\left(l-1\right)}{\mathbf{W}}_{Q}^{\tau },{\mathbf{K}}^{\left(\tau \right)}={\mathbf{H}}_{\tau }^{\left(l-1\right)}{\mathbf{W}}_{K}^{\tau },{\mathbf{V}}^{\left(\tau \right)}\\&={\mathbf{H}}_{\tau }^{\left(l-1\right)}{\mathbf{W}}_{V}^{\tau },\end{aligned}$$

where ${\mathbf{W}}_{Q}^{\tau },{\mathbf{W}}_{K}^{\tau },{\mathbf{W}}_{V}^{\tau }\in {\mathbb{R}}^{{d}_{h}\times {d}_{h}}$. The projected vectors are reshaped into $H$ attention heads, each with dimension ${d}_{k}$.

For an edge $s\to t$, let $s$ be the source node, $t$ be the target node, and $r\in \mathrm{0,1}$ be the edge type, where $r=0$ denotes a gene-to-cell edge and $r=1$ denotes a cell-to-gene edge. For each attention head $h$, scFormer applies relation-specific transformations to the key and value vectors:

13 $$\begin{array}{c}{\widetilde{\mathbf{k}}}_{s}^{r,h}={\mathbf{W}}_{ATT}^{r,h}{\mathbf{k}}_{s}^{h},{\widetilde{\mathbf{v}}}_{s}^{r,h}={\mathbf{W}}_{MSG}^{r,h}{\mathbf{v}}_{s}^{h},\end{array}$$

where ${\mathbf{W}}_{ATT}^{r,h},{\mathbf{W}}_{MSG}^{r,h}\in {\mathbb{R}}^{{d}_{k}{\times d}_{k}}$. A relation-specific prior ${\mu}_{r,h}$ is also learned for each edge type and attention head**.**

The unnormalized attention score from source node $s$ to target node $t$ under edge type $r$ and head $h$ is computed as:

14 $$\begin{array}{c}{e}_{ts}^{r,h}=\frac{{\mathbf{q}}_{t}^{h}\cdot {\widetilde{\mathbf{k}}}_{s}^{r,h}}{\sqrt{{d}_{k}}}{\mu}_{r,h}.\end{array}$$

The attention coefficient is then obtained by applying Softmax over all incoming edges of the same target node $t$, separately for each attention head:

15 $$\begin{array}{c}{\upalpha}_{ts}^{r,h}=\frac{\mathrm{e}\mathrm{x}\mathrm{p}({e}_{ts}^{r,h})}{{\sum}_{s{\prime}\in \mathcal{N}(t)}{\mathrm{e}\mathrm{x}\mathrm{p}(e}_{ts{\prime}}^{r,h})}.\end{array}$$

Thus, the Softmax normalization is performed across source nodes connected to the same target node, rather than across all nodes or all edges in the graph.

The message passed from source node $s$ to target node $t$ is computed for each head as:

16 $$\begin{array}{c}{\mathbf{m}}_{ts}^{r,h}={\upalpha}_{ts}^{r,h}{\widetilde{\mathbf{v}}}_{s}^{r,h}.\end{array}$$

Messages from all heads are concatenated, and incoming messages are summed over the neighborhood of the target node:

17 $$\begin{array}{c}{\mathbf{m}}_{t}^{l}=\sum\limits_{s\in \mathcal{N}(t)}{\mathrm{C}\mathrm{o}\mathrm{n}\mathrm{c}\mathrm{a}\mathrm{t}}_{1}^{H}({\mathbf{m}}_{ts}^{r,h}).\end{array}$$

The aggregated message is then passed through a node-type-specific output projection, followed by dropout, a learnable skip connection, and layer normalization:

18 $$\begin{array}{c}{\mathbf{h}}_{t}^{l}={\mathrm{L}\mathrm{a}\mathrm{y}\mathrm{e}\mathrm{r}\mathrm{N}\mathrm{o}\mathrm{r}\mathrm{m}}_{{\tau}_{t}}\left({\beta}_{{\tau}_{t}}{\mathbf{W}}_{O}^{{\tau}_{t}}\mathrm{G}\mathrm{E}\mathrm{L}\mathrm{U}\left({\mathbf{m}}_{t}^{l}\right)+\left(1-{\beta}_{{\tau}_{t}}\right){\mathbf{h}}_{t}^{l-1}\right),\end{array}$$

where ${\tau}_{t}$ is the type of target node $t$, ${\mathbf{W}}_{O}^{{\tau}_{t}}\in {\mathbb{R}}^{{d}_{h}\times {d}_{h}}$ is the node-type-specific output projection, and ${\beta}_{{\tau}_{t}}$ is a learnable skip coefficient. Stacking multiple HGT layers yields the final cell embeddings ${\mathbf{H}}_{c}^{\mathrm{f}\mathrm{i}\mathrm{n}\mathrm{a}\mathrm{l}}$ and gene embeddings ${\mathbf{H}}_{g}^{\mathrm{f}\mathrm{i}\mathrm{n}\mathrm{a}\mathrm{l}}$, which are subsequently used for clustering and reconstruction.

### Training of scFormer

During training, each mini-batch cell-gene subgraph generated by the aforementioned subsampling strategy was passed through the HGT network. The batch-specific node features were propagated through HGT layers to compute cell embeddings and gene embeddings. For a mini-batch containing the sampled cell set $B$ and the selected gene set ${\Gamma}_{B}$, the resulting cell and gene embeddings are denoted as follows:

19 $$\begin{array}{c}{\mathbf{H}}_{c}\in {\mathbb{R}}^{\left|B\right|\times {d}_{h}},{\mathbf{H}}_{g}\in {\mathbb{R}}^{\left|{\Gamma}_{B}\right|\times {d}_{h}},\end{array}$$

where ${d}_{h}$ denotes the latent dimension. The standardized matrix $\mathbf{Z}$ was used for node input features, whereas the non-negative matrix $\mathbf{X}$ was retained as the reconstruction target. These embeddings were optimized using a multi-component objective consisting of a KL-based reconstruction regularizer, a pseudo-label-based clustering loss, an intra-cluster compactness term, and an optional batch-correction term.

#### KL divergence loss for information preservation

The reconstruction objective was computed using the non-negative mini-batch expression submatrix ${\mathbf{X}}_{B,{\Gamma}_{B}}$, rather than the standardized Z-score matrix $\mathbf{Z}$. Given the learned cell embeddings ${\mathbf{H}}_{{\boldsymbol{c}}}$ and gene embeddings ${\mathbf{H}}_{{\boldsymbol{g}}}$, scFormer decoded cell-gene affinities using inner products between cell and gene embeddings:

20 $$\begin{array}{c}{S}_{c\to g}={\mathbf{H}}_{c}{\mathbf{H}}_{g}^{\top },{S}_{g\to c}={\mathbf{H}}_{g}{\mathbf{H}}_{c}^{\top }.\end{array}$$

The decoded affinity matrices and the corresponding non-negative expression targets were converted into probability distributions by row-wise softmax over matched dimensions:

21 $$\begin{aligned}&{P}_{c\to g}={\mathrm{softmax}}_{g}\left({X}_{B,{\Gamma}_{B}}\right),{Q}_{c\to g}\\& ={\mathrm{softmax}}_{g}\left({S}_{c\to g}\right),\end{aligned}$$

22 $$\begin{aligned}&{P}_{g\to c}={\mathrm{softmax}}_{c}\left({X}_{B,{\Gamma}_{B}}^{\top }\right),{Q}_{g\to c}\\&={\mathrm{softmax}}_{c}\left({S}_{g\to c}\right).\end{aligned}$$

The KL divergence regularizer was then defined as:

23 $$\begin{array}{c}{\mathcal{L}}_{KL}={D}_{KL}\left({P}_{c\to g}\parallel {Q}_{c\to g}\right)+{D}_{KL}\left({P}_{g\to c}\parallel {Q}_{g\to c}\right),\end{array}$$

where ${D}_{KL}\left(P\parallel Q\right)=\frac{1}{N}{\sum}_{i=1}^{N}{P}_{i}\mathrm{log}\frac{{P}_{i}}{{Q}_{i}}$ quantifies the divergence between two distributions $P$ (original) and $Q$ (reconstructed).

This KL term was used as an auxiliary distribution-matching regularizer, not as a likelihood model for raw sparse counts. Library-size effects were addressed before training by normalization in $\mathbf{X}$. Because this term is not formulated as a count-generative likelihood, scFormer does not explicitly model zero inflation. Zero entries are retained in the non-negative matrix $\mathbf{X}$ and contribute to the softmax-normalized target distributions within each mini-batch. After softmax normalization, the reconstruction term compares relative cell-gene association patterns within each mini-batch rather than absolute count magnitudes. Importantly, the softmax normalization was applied to the mini-batch expression submatrix defined by the sampled cells $B$ and the selected gene set ${\Gamma}_{B}$, rather than to the full transcriptome. Therefore, the KL term regularizes relative cell-gene association patterns within the selected mini-batch subgraph after graph construction, rather than driving gene selection from all detected genes. Because the reconstruction target is $\mathbf{X}$, negative standardized Z-score values are not used in the KL objective. Thus, the loss avoids applying KL divergence directly to negative standardized values while still encouraging the learned embeddings to preserve expression-derived cell-gene relationships.

#### Cross-entropy loss for cell type discrimination

Initial pseudo-labels were generated once before model training using Leiden clustering on the normalized expression matrix $\mathbf{X}$. These pseudo-labels were kept fixed during training and used as weak supervision to stabilize the learned cell embeddings. Resolution parameters were chosen according to dataset size, with 0.2 for datasets containing < = 500 cells, 0.5 for datasets containing 500–5,000 cells, and 0.8 for datasets containing > 5,000 cells when no custom value was provided. Neighborhood graphs for Leiden initialization used 5, 10, or 15 nearest neighbors for small, medium, or large datasets, respectively, unless a dataset-specific setting was specified.

The clustering loss was computed using label smoothing between the pseudo-labels and the model-predicted cell probabilities:

24 $$\begin{array}{c}{\mathcal{L}}_{cluster}=-\frac{1}{\left|B\right|}{\sum}_{i\in B}{\sum}_{k=1}^{{K}_{c}}{\widetilde{y}}_{ik}log{p}_{ik},\end{array}$$

where ${K}_{c}$ is the number of pseudo-label classes in the training set, ${\widetilde{y}}_{ik}$ denotes the smoothed pseudo-label distribution, and ${p}_{ik}$ denotes the predicted probability that cell $i$ belongs to class $k$.

#### Intra-cluster compactness term

To encourage compact cell embeddings within each pseudo-labeled group, scFormer computed the mean cosine similarity among cells assigned to the same pseudo-label within each mini-batch. For each class $k$ represented in the mini-batch, let ${C}_{k}$ denote the set of cells assigned to that class. The intra-cluster similarity score was defined as:

25 $$\begin{array}{c}{\mathcal{S}}_{intra}=\sum\limits_{k:\left|{C}_{k}\right|>1}\frac{1}{{\left|{C}_{k}\right|}^{2}}\sum\limits_{i,j\in {C}_{k}}\frac{{\mathbf{H}}_{c}^{\left(i\right)}\cdot {\mathbf{H}}_{c}^{\left(j\right)}}{\Vert {\mathbf{H}}_{c}^{\left(i\right)}\Vert \Vert {\mathbf{H}}_{c}^{\left(j\right)}\Vert }.\end{array}$$

This term is a similarity score rather than a positive penalty. It was therefore subtracted from the total objective, consistent with maximizing intra-cluster compactness during optimization.

#### Batch correction loss (optional)

When batch correction was enabled, scFormer added a batch-correction term computed from the cell embeddings in the current mini-batch. This term consisted of a distribution-alignment component and a local-structure component. For each batch $b$ represented in the mini-batch, the mean embedding vector ${\mu}_{b}$ and variance vector ${v}_{b}$ were computed across cells from that batch. The distribution-alignment term penalized variation in these batch-level embedding moments:

26 $$\begin{array}{c}{\mathcal{L}}_{distribution}={\mathrm{m}\mathrm{e}\mathrm{a}\mathrm{n}}_{l}{\mathrm{V}\mathrm{a}\mathrm{r}}_{b}\left({\mu}_{b,l}\right)+0.5{\mathrm{m}\mathrm{e}\mathrm{a}\mathrm{n}}_{l}{\mathrm{V}\mathrm{a}\mathrm{r}}_{b}\left({v}_{b,l}\right).\end{array}$$

The local-structure component was computed from Euclidean distances between cells and their nearest neighbors in the embedding space. The number of neighbors was set to ${k}_{s}=min(15,\left|B\right|-1)$:

27 $$\begin{array}{c}{\mathcal{L}}_{structure}=\frac{1}{\left|B\right|}\sum\limits_{i\in B}\frac{1}{{k}_{s}}\sum\limits_{j=1}^{{k}_{s}}{\Vert {H}_{c}^{i}-{H}_{c}^{{n}_{i}(j)}\Vert }_{2},\end{array}$$

where ${n}_{i}(j)$ denotes the index of the j-th nearest neighboring cell of cell $i$ among the other cells in the same mini-batch, based on Euclidean distance in the cell embedding space. The optional batch-correction loss was:

28 $$\begin{array}{c}{\mathcal{L}}_{batch}={\mathcal{L}}_{distribution}+0.5{\mathcal{L}}_{structure}.\end{array}$$

#### Total loss function

The overall training objective used fixed weights for all reported experiments:

29 $$\begin{array}{c}{\mathcal{L}}_{total}={\mathcal{L}}_{KL}+{\mathcal{L}}_{cluster}-{\mathcal{S}}_{intra}+{I}_{batch}\cdot 0.5{\mathcal{L}}_{batch}.\end{array}$$

where ${I}_{batch}$ equals 1 when batch correction is enabled and 0 otherwise. Thus, without batch correction, the optimized objective is ${\mathcal{L}}_{KL}+{\mathcal{L}}_{cluster}-{\mathcal{S}}_{intra}$. With batch correction enabled, the optional batch term is added with coefficient 0.5. No additional tuned loss weights were used in the main experiments.

Model parameters were updated using the AdamW optimizer with learning rate $5\times {10}^{-4}$ and weight decay 0.1. Dropout was set to 0.3 in the HGT network, and label smoothing was set to 0.1 for the pseudo-label classification term. Training was run for a fixed 100 epochs in the main experiments, and the final model state was used for downstream embedding extraction and clustering. No validation split, validation-based early stopping, or validation-based model selection was used.

This training protocol reflects the intended use of scFormer as a dataset-specific representation-learning method for single-cell and spatial transcriptomic analyses. The model is trained separately for each dataset to learn embeddings for downstream analysis, rather than being trained as a supervised predictor intended for direct transfer to unseen datasets. To reduce unstable optimization and degenerate memorization, training combined sparse cell-gene graph construction, dropout, AdamW weight decay, label smoothing, and the multi-component objective described above.

### Inference of cell clusters

Following model training, we apply the robustly trained classifier to the entire cellular graph to assign each cell to its predicted cluster. The model maintains performance across the complete dataset by leveraging a union of subgraphs that encompasses all cells. The trained network produces HGT-derived cell embeddings, which are then mapped to per-cell assignment scores via a learnable assignment function. Final cluster labels are obtained by selecting the highest-scoring cluster (argmax), yielding predicted cluster assignments across the dataset. This approach yields a comprehensive set of predicted cluster labels across the entire dataset, facilitating downstream biological interpretation and analysis.

### Simulated data

To construct a systematic benchmarking dataset for evaluating rare-cell identification by scFormer and competing methods, synthetic scRNA-seq datasets were generated using Splatter (Zappia et al. 2017) (v1.30.0) across a predefined multidimensional parameter grid. A full-factorial design was used to vary three differential expression (DE)-related parameters: de.prob (0.1, 0.3, 0.5, 0.7, 0.9), de.facLoc (0.1, 0.3, 0.5, 0.7, 0.9), and de.facScale (0.2, 0.4, 0.6, 0.8, 1.0), resulting in 125 unique parameter combinations. For each combination, one dataset containing 2,500 cells and 5,000 genes was simulated. Cells were assigned to a major population (Group1) and a rare population (Group2) at a 99:1 ratio to model extreme class imbalance typical of rare-population settings.

Simulated count matrices were processed using a standardized preprocessing workflow. Specifically, counts were log-transformed as log2(counts +1) for methods requiring normalized inputs; for methods operating on raw counts, the original simulated counts were used as input. No label-informed gene filtering or post hoc modification of the simulated gene set was performed, ensuring that all methods were evaluated on the same underlying simulated expression matrices.

To quantify the transcriptomic separability between the major and rare populations across parameter combinations, a separation metric was computed for each dataset. Principal component analysis (PCA) was applied to the expression matrix, and the centroid of each population was calculated along the first principal component (PC1). Dataset separation was defined as the absolute difference between the two centroids on PC1. This separation metric was used to characterize a continuous difficulty gradient across the simulated datasets for downstream performance stratification.

### Comparison with other methods

To evaluate the performance of scFormer in the task of rare-cell identification, a systematic comparison was conducted against seven representative rare-cell identification methods. The selected competing methods, including CellSIUS, FiRE, GapClust, GiniClust3, RaceID3, scCAD, and SCMER, cover rarity-scoring, differential-expression, clustering-based, and feature-learning strategies, and were selected because they could be applied consistently across the simulated and real scRNA-seq benchmarks. Methods that require additional modalities, spatial coordinates, or do not directly produce rare-cell assignments were not included in the rare-cell F1 benchmark, but relevant batch-correction methods were evaluated separately in the multi-sample PBMC analysis. To ensure reproducibility and to reduce inadvertent bias introduced by dataset-specific tuning, we followed the parameterization recommended by the original publications and official documentation for each method, and we applied a consistent evaluation protocol across all datasets. Method parameters were not optimized against the evaluation labels; instead, key settings were fixed before performance evaluation according to the original publications, official tutorials, or package defaults. The complete software versions and parameter settings are provided in Supplementary Table 1. The datasets were processed and evaluated following the official tutorials for each respective method. The performance of each algorithm was quantitatively compared using standard classification metrics, including precision, recall, and F1 score.

Furthermore, to assess the capability of scFormer in batch effect correction, a comparative analysis was performed against prominent batch correction methods, namely Harmony, BBKNN, MNN Correct, and ComBat. Batch-correction baselines were run using standardized implementations with commonly used recommended settings. For the Harmony-Leiden pipeline used as the two-stage integration and clustering baseline in Fig. 4, Harmony was applied to PCA embeddings using batch labels, followed by nearest-neighbor graph construction on Harmony-corrected embeddings and Leiden clustering. To assess the effect of Leiden resolution, we performed a resolution sweep across 0.2, 0.4, 0.6, 0.8, 1.0, 1.2, 1.5, 2.0, 2.5, and 3.0. At each resolution, predicted rare cells were defined using the same cluster-abundance criterion as in the main analysis, namely cells assigned to clusters comprising ≤ 5% of the dataset. The complete resolution-sweep results are provided in Supplementary Table 2.

### Rare population definition

For real datasets, we used the original study annotations as reference cell-type annotations. A reference cell type was defined as *rare* if its prevalence within the dataset was ≤ 5% (default threshold r = 5%). For each method, predicted rare cells were defined as cells belonging to clusters whose abundance was ≤ 5% of all cells in that dataset. We then computed cell-level precision, recall, and F1 score by comparing predicted rare cells against reference rare cells (rare vs non-rare). These published annotations were used as reference labels rather than definitive biological ground truth. Therefore, precision, recall, and F1 score in real-data benchmarks quantify concordance with the original study annotations at the rare-versus-non-rare level.

### Evaluation index

For real scRNA-seq datasets, we evaluated annotation-level rare-cell recovery by calculating precision, recall, and F1 score against the reference rare labels defined from the original study annotations. Precision quantifies the proportion of cells predicted as rare that were also labeled as rare in the reference annotations, calculated as:

30 $$\begin{array}{c}Precision=\frac{TP}{TP+FP},\end{array}$$

where *TP* (true positives) denotes cells predicted as rare that were also labeled as rare in the reference annotations, and *FP* (false positives) denotes cells predicted as rare but labeled as non-rare in the reference annotations. High precision ensures minimal false positives, enhancing the reliability of detecting biologically significant cell types.

Recall, or sensitivity, measures the proportion of reference-labeled rare cells recovered as rare by each method, defined as:

31 $$\begin{array}{c}Recall=\frac{TP}{TP+FN},\end{array}$$

where FN (false negatives) denotes cells labeled as rare in the reference annotations but not recovered as rare by the method. Given the low abundance of reference rare cells in scRNA-seq datasets, high recall is important for reducing missed reference-annotated rare populations.

The F1 score was the harmonic mean of precision and recall, calculated as:

32 $$\begin{array}{c}\mathrm{F1}\ score=2\times \frac{Precision\cdot Recall}{Precision+Recall},\end{array}$$

balances these metrics to provide a robust evaluation, particularly in class-imbalanced scenarios. In real-data benchmarks, higher precision, recall, and F1 scores indicate stronger concordance with published reference rare-cell annotations. Under this annotation-level evaluation, scFormer showed stronger overall concordance than competing methods across diverse scRNA-seq datasets.

Additionally, we employed the integration local inverse Simpson’s index (iLISI) and batch entropy to assess scFormer’s batch correction performance in integrating multi-batch scRNA-seq datasets. These metrics evaluate the model’s ability to mitigate batch effects while preserving biological heterogeneity, ensuring reliable downstream analyses such as differential expression or trajectory inference. The iLISI measures local batch mixing by computing the inverse of the Simpson’s diversity index within the KNN graph of each cell, expressed as:

33 $$\begin{array}{c}iLISI=\frac{1}{{\sum}_{b}{p}_{b}^{2}},\end{array}$$

where ${p}_{b}$ is the proportion of cells from batch *b* in the neighborhood. Higher *iLISI* values indicate better batch integration, reflecting a uniform distribution of cells across batches in local structures.

Batch entropy quantifies the global uniformity of batch distribution, calculated as:

34 $$\begin{array}{c}Entropy=-{\sum}_{b}{p}_{b}\cdot log({p}_{b}),\end{array}$$

where ${p}_{b}$ represents the proportion of cells from batch *b* in the dataset. Elevated entropy values signify reduced batch-related biases, indicating effective batch correction.

### Post hoc spatial enrichment analysis

For the sci-Space slide_4E analysis, spatial coordinates were not used as input to scFormer, but were used only for post hoc evaluation. Spatial self-enrichment was quantified as the fraction of same-cluster cells among the 10 nearest spatial neighbors for each cell and compared with 10,000 size-matched random permutations. Empirical *p* values were adjusted using the Benjamini–Hochberg procedure. Concordance with published sci-Space annotations was evaluated using hypergeometric enrichment tests, and marker/module enrichment was quantified by comparing mean expression in each target cluster with all remaining cells.

### Statistics and reproducibility

This study did not employ statistical methods to predetermine sample size, nor were randomization or blinding applied during experimental design or result evaluation. No data were excluded from the analysis beyond standardized data quality control measures. To ensure reproducibility of simulated data generation, a fixed random seed was set using splatter (Zappia et al. 2017) (v.1.30.0). Differentially expressed genes (DEGs) were identified using the FindMarkers function from the Seurat R package (Hao et al. 2024) (v.5.3.0), employing a two-sided Wilcoxon rank sum test with a false discovery rate (FDR) threshold of 0.05 and an inter-group absolute fold-change threshold of 1.5. Fold-change values were calculated based on the mean expression levels of each gene between groups. *p* values were adjusted using Bonferroni correction to account for multiple testing across the total number of genes in the dataset.

## Results

### Overview of the scFormer framework

Instead of relying on cell–cell homophily, scFormer encodes rare-cell specificity directly into a cell-gene heterogeneous graph, allowing rare transcriptional programs to propagate through the network without being diluted by dominant cell types. scFormer is a graph-based computational framework designed to detect rare cell populations by encoding biological priors directly into the underlying topology. Rather than treating all expressed genes equivalently, scFormer is motivated by the premise that broadly expressed genes provide limited discriminatory power for resolving rare states. Instead, it prioritizes genes exhibiting high cell-specific expression, treating them as informative anchors for distinguishing rare populations.

**Fig. 1:**
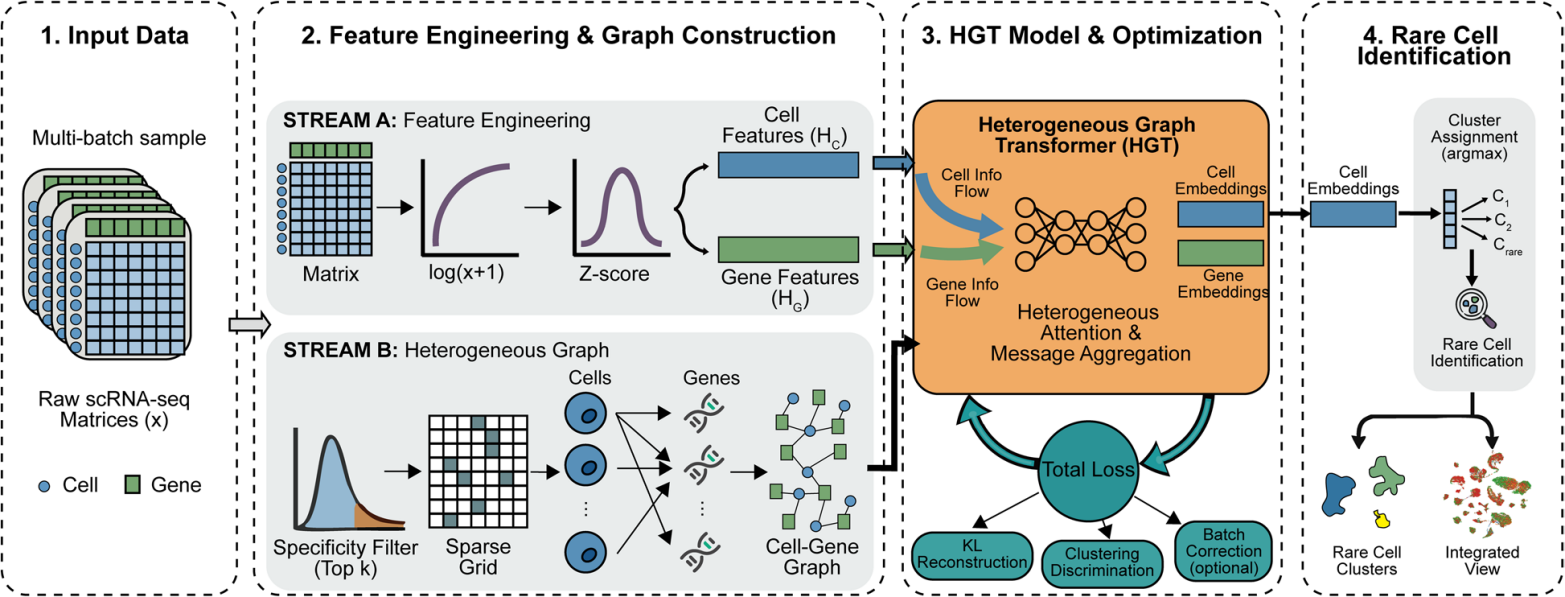
Overview of the scFormer framework. The workflow is divided into four sequential stages: (1) Input Data: raw scRNA-seq count matrices from one or more batches are processed to generate normalized cell- and gene-level features. (2) Feature Engineering & Graph Construction: This stage processes data through two parallel streams. The first stream (top) performs normalization to generate cell and gene feature matrices (${\mathrm{H}}_{\mathrm{C}}$ and ${\mathrm{H}}_{\mathrm{G}}$). The second stream (bottom) constructs a cell-gene heterogeneous graph by filtering for specificity (Top Z-scores) to link cells with specific marker genes. (3) HGT Model & Optimization: The constructed heterogeneous graph and preprocessed features are fed into the HGT model. Through heterogeneous attention and message aggregation, the model learns low-dimensional embeddings, optimized by a total loss function (KL reconstruction, clustering discrimination, and optional batch correction). (4) Rare-Cell Identification: Cluster assignments are obtained by applying a learnable assignment function to the HGT-derived cell embeddings. Each cell is assigned to the cluster with the highest predicted score

As shown in Fig. 1, the framework comprises four stages: input scRNA-seq matrices, feature engineering and heterogeneous graph construction, HGT-based representation learning with multi-objective optimization, and rare-cell identification.

Rather than simply preprocessing data to reduce noise, scFormer transforms the normalized expression matrix into a structured graph that highlights unique biological signals. While the initial phase handles raw input data (Step 1) and standard normalization (Step 2, top pathway), scFormer’s critical innovation lies in its specificity-driven topology construction. Specifically, scFormer employs a quantitative selection strategy (Step 2, bottom pathway) where cell-gene connections are established only for genes exhibiting high expression and statistically significant specificity (calculated via Z-scores) within a given cell. This approach ensures that the graph captures biologically relevant genes that are highly expressed in specific cell types but underexpressed elsewhere, embedding critical biological priors into the graph’s topological structure. Unlike traditional graph neural networks (GNNs) that rely on k-nearest neighbor (KNN) graphs to model cell–cell relationships, which often assume homophily and connect cells with similar features (Levine et al. 2015; Liu et al. 2025; Yu et al. 2023), scFormer uses genes as an intermediary bridge to link cells. This gene-mediated approach overcomes the limitations of homophily-based graphs, which may overlook heterogeneous relationships, and better captures complex biological interactions by leveraging gene expression patterns to connect cells with diverse functional roles.

The constructed cell-gene heterogeneous graph is processed by an HGT model (Step 3), which captures complex cell-gene interactions through node- and edge-specific operations tailored for heterophily. Unlike conventional GNNs that assume homophily, scFormer’s HGT is designed to handle heterophily, accommodating diverse cell-gene relationships where connected nodes may represent distinct biological entities. The model is optimized via a unified multi-objective loss function that primarily enforces accurate gene expression reconstruction and maximizes clustering consistency. While optimized for single-sample analysis, the framework offers the flexibility to incorporate an auxiliary loss term for batch effect correction, extending its applicability to multi-sample datasets without compromising its core performance. Finally, during inference (Step 4), cluster assignments are obtained by applying a learnable assignment function to the HGT-derived cell embeddings. Each cell is assigned to the cluster with the highest assignment score, enabling downstream identification of rare populations based on cluster abundance and marker enrichment.

### scFormer demonstrates high robustness and accuracy in simulated benchmarks

To systematically assess sensitivity to rare populations, we generated 125 synthetic datasets using the Splatter package (Zappia et al. 2017). Each dataset comprised 2,500 cells and 5,000 genes, with two clusters present at a 99:1 proportion. This extreme imbalance was designed to emulate rare populations comprising ~1% of cells, a regime commonly encountered when profiling low-frequency immune subsets or small stem/progenitor niches. To probe robustness across varying levels of difficulty, simulation settings were varied to generate datasets spanning highly distinct to minimally separable cell populations. Dataset separation was quantified as the distance between cluster centroids along the first principal component (Supplementary Fig. 2).

**Fig. 2:**
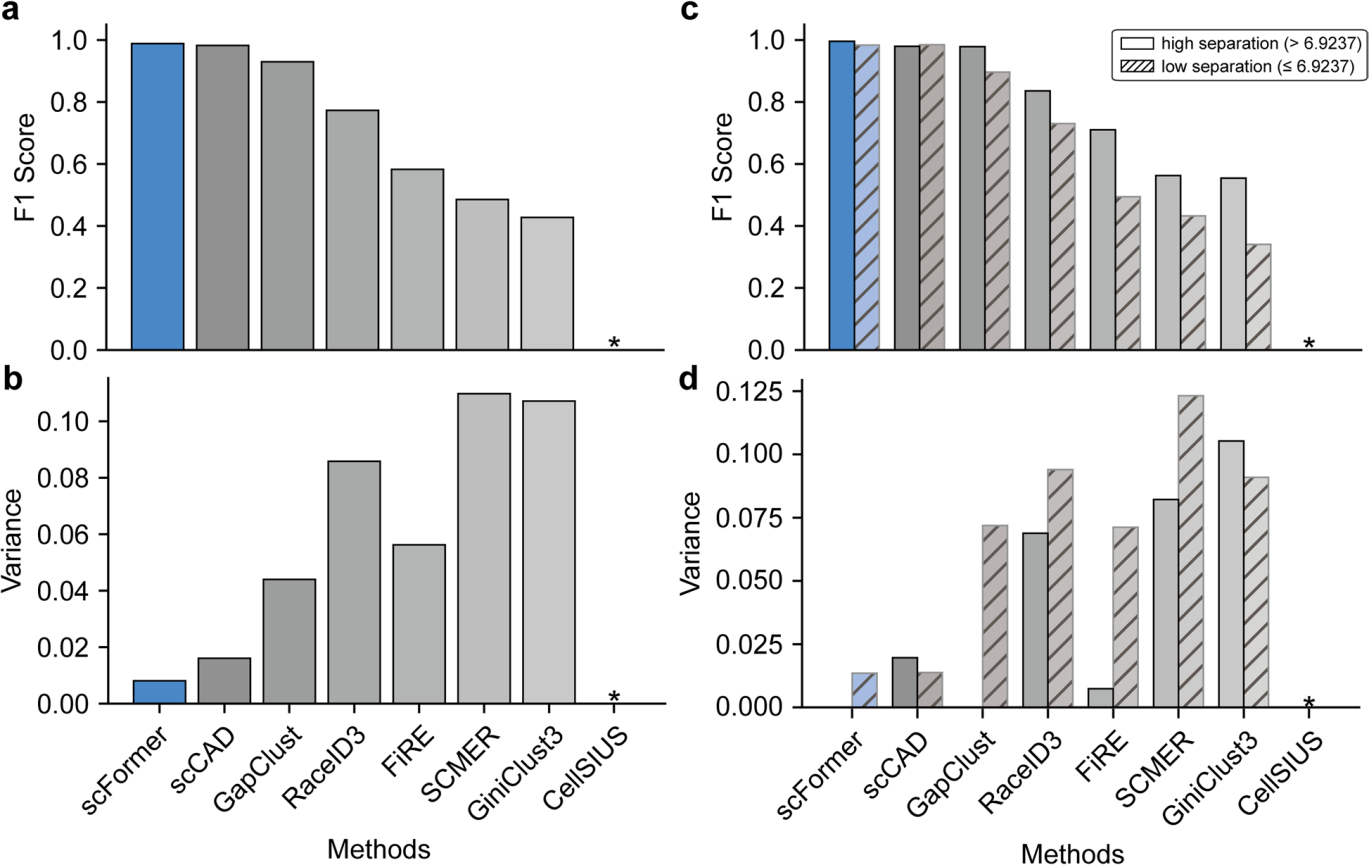
scFormer demonstrates superior performance in identifying rare-cell clusters across varying dataset difficulties. **a** F1 score comparison of scFormer and seven competing methods (scCAD, GapClust, RaceID3, FiRE, SCMER, GiniClust3, CellSIUS) across 125 simulated datasets with 2,500 cells, 5,000 genes, and extreme cluster imbalance (99:1 ratio). scFormer achieves the highest average F1 score (0.988), followed by scCAD (0.982) and GapClust (0.930). **b** Variance in F1 scores across all datasets, showing scFormer exhibits the lowest variance (0.008), indicating superior stability and robustness to parameter variations. **c** Performance comparison stratified by separation difficulty. Datasets were divided using the 10th percentile (6.9237) as the cutoff: high-separation (solid bars, separation index > 6.9237) and low-separation (striped bars, separation index ≤ 6.9237) conditions. **d** Variance comparison between high- and low-separation groups, demonstrating that scFormer maintains exceptional stability even under challenging conditions with minimal transcriptomic differences between cell clusters. * indicates that CellSIUS yielded F1 scores of zero across all datasets

scFormer was benchmarked against seven representative methods (CellSIUS, FiRE, GapClust, GiniClust3, RaceID3, scCAD, and SCMER). Performance was evaluated using precision, recall, and F1 score (Supplementary Dataset 1). Across all simulations, scFormer achieved the highest overall F1 score (0.988) and exhibited strikingly low variance, indicating strong robustness to extreme imbalance and heterogeneous signal strengths (Fig. 2a-b).

To explicitly test biologically challenging scenarios, where rare populations differed only subtly from abundant populations, datasets were stratified by separation difficulty using the centroid distance metric. The 10th percentile of the separation distribution (6.9237) was used to distinguish low- from high-separation scenarios. Low-separation settings reflect cases such as closely related cell subtypes, subtle activation states, or continuous state transitions with small transcriptional effect sizes, which are known to confound rare-cell identification by reducing separability while maintaining strong abundance imbalance.

Under this stratification, scFormer maintained high and stable performance in both low- and high-separation conditions (Fig. 2c-d). While several baseline methods exhibited marked performance degradation and increased variability in low-separation datasets, scFormer remained robust, supporting reliable recovery of rare clusters even when inter-cluster differences were minimal. Notably, scCAD showed comparable accuracy to scFormer in some settings (Fig. 2c) but displayed weaker stability across conditions (Fig. 2d). Together, these results highlight scFormer’s advantage in jointly optimizing accuracy and robustness, which is critical for sensitively detecting biologically meaningful rare subpopulations in complex single-cell datasets. A sensitivity analysis of the graph-sparsity parameter $K$ further showed that the default value *K* = 20 reached a near-performance plateau while avoiding the additional runtime and graph-size increase observed with larger $K$ values (Supplementary Fig. 1).

### scFormer achieves state-of-the-art performance on real datasets

To evaluate scFormer under real biological heterogeneity, performance was assessed on 18 publicly available scRNA-seq datasets spanning diverse tissues and biological contexts, including immune (PBMC), epithelial (airway), nervous system (brain and retina), and multiple organ and disease settings (kidney, liver, pancreas, breast, tonsil, and renal cell carcinoma), with dataset sizes ranging from 313 (Deng et al. 2014) to 49,147 (Hao et al. 2021) cells (Supplementary Table 3). Rare populations were defined from the original study annotations as cell types with prevalence ≤ 5% within each dataset and used as reference labels. Because annotation granularity differs across studies, these labels were treated as reference annotations for benchmarking rather than absolute ground truth. Unless otherwise specified, predicted rare cells were defined as cells in clusters with abundance ≤ 5%.

**Fig. 3:**
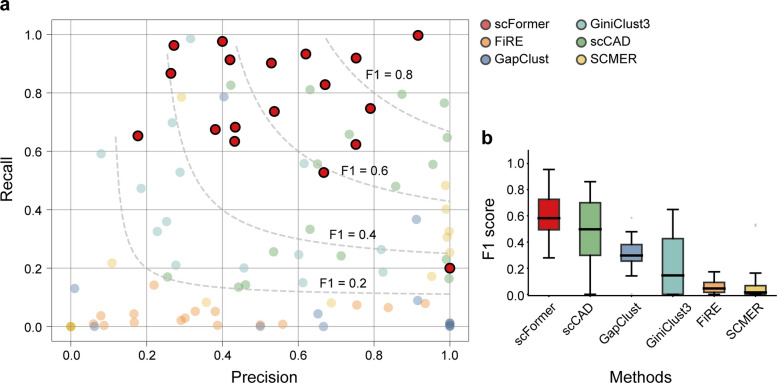
Annotation-level performance evaluation of scFormer against state-of-the-art methods across 18 scRNA-seq datasets. **a** Precision-recall scatter plot showing the performance distribution of six methods (scFormer, GiniClust3, FIRE, scCAD, GapClust, and SCMER) across all datasets. Each point represents one dataset, with dashed gray lines indicating F1 score contours (0.2, 0.4, 0.6, 0.8). **b** Box plot comparison of F1 scores across all methods, displaying median values, interquartile ranges, and performance distribution. Statistical significance and detailed dataset information are provided in Supplementary Dataset 2

Across datasets, scFormer showed the best overall annotation-level rare-cell recovery performance (Fig. 3a). In the precision-recall landscape, scFormer most frequently occupied the high-precision/high-recall regime, indicating an improved balance between sensitivity and false discovery compared with competing methods. Aggregated across datasets, scFormer achieved the highest mean F1 score (0.595), corresponding to a 22.1% improvement over scCAD (0.488) and a 93.8% improvement over GiniClust3 (0.307) (Fig. 3b). Consistent with these averages, the F1 score distribution of scFormer exhibited the highest median with a comparatively tight interquartile range, supporting strong concordance with published reference annotations and stable behavior across heterogeneous tissues and study designs.

Representative case studies further illustrate scFormer’s robustness at the dataset level. In a mouse retina dataset (Dou et al. 2022), scFormer achieved an F1 score of 0.955, substantially higher than scCAD (0.649) and GiniClust3 (0.282). In a more challenging setting with pronounced cellular heterogeneity, the human renal cell carcinoma dataset (Zhang et al. 2021), scFormer remained the top-performing method with an F1 score of 0.279, exceeding scCAD (0.207) and GiniClust3 (0.267). Together, these results support scFormer as a robust approach for recovering reference-annotated rare populations across tissues and study designs.

### scFormer balances batch correction and rare-cell recovery during multi-sample integration

To evaluate scFormer for multi-sample integration, performance was assessed with respect to batch effect correction, while considering the need to preserve subtle signals relevant to rare-cell identification after integration. A PBMC dataset comprising 30,669 cells and 14,087 genes across four batches was used, and cell-type annotations from the original study were treated as the reference for quantitative evaluation.

**Fig. 4:**
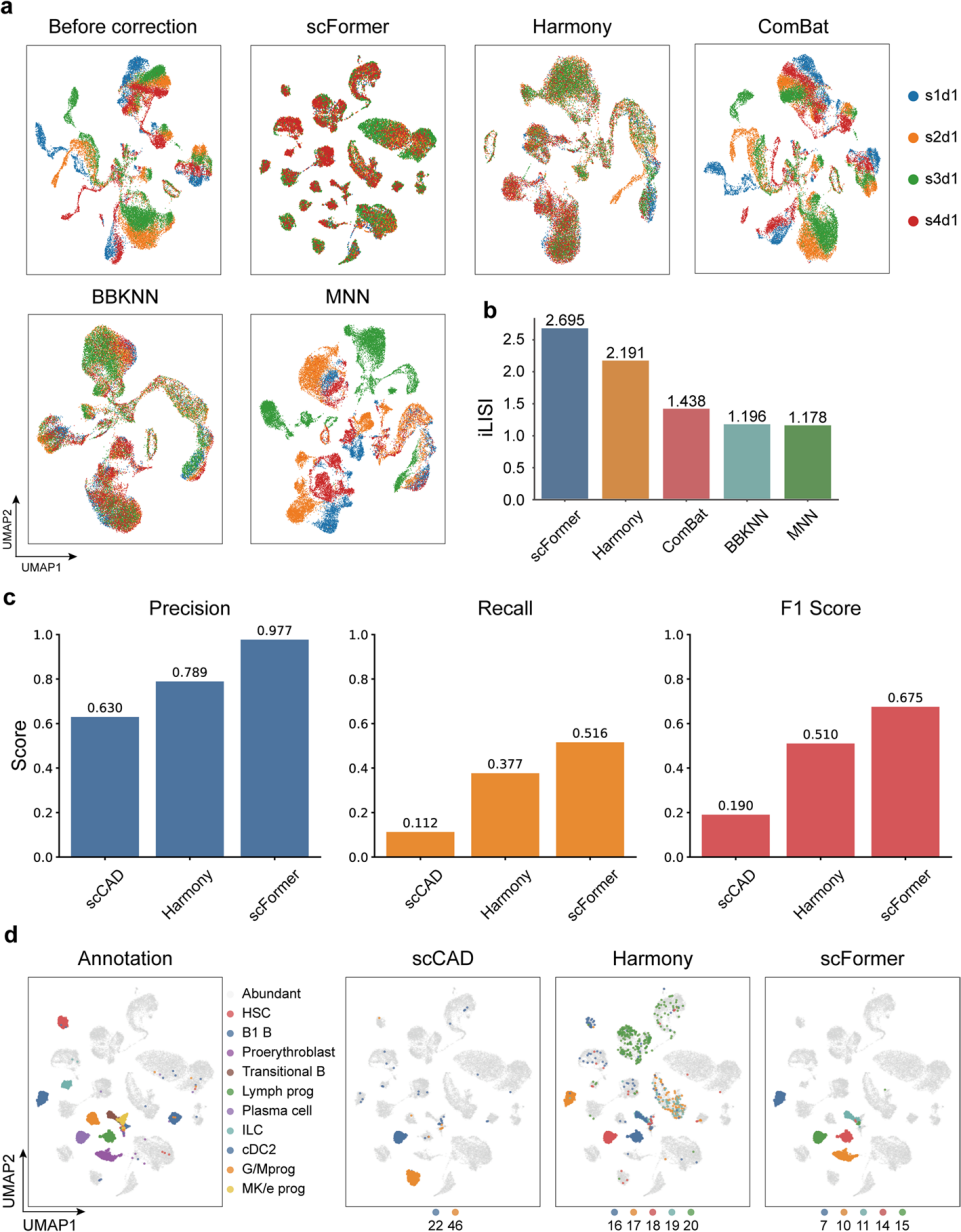
scFormer simultaneously corrects batch effects and identifies rare cell populations. **a** UMAP plots illustrating batch integration performance in a PBMC dataset (30,669 cells across four batches). Visualizations compare the uncorrected data with results from scFormer, Harmony, ComBat, BBKNN, and MNN. Colors denote the batch of origin for each cell (s1d1-s4d1, where s indicates sample and d indicates donor; all batches are four samples from donor 1). **b** Quantitative evaluation of batch correction performance. scFormer achieves the highest iLISI score (2.695), exceeding Harmony and other benchmarked methods, which signifies more effective batch integration. **c** Precision, recall, and F1 scores for rare-cell identification using scFormer, scCAD and the Harmony-Leiden pipeline. For Harmony-Leiden, Harmony was used for batch correction followed by Leiden clustering, with clusters ≤ 5% of cells designated as rare. Sensitivity to Leiden resolution was evaluated separately across resolutions from 0.2 to 3.0, with the complete results provided in Supplementary Table 2. **d** UMAP projections of 30,669 PBMC cells colored by the original annotations (leftmost) alongside the rare clusters identified by scCAD (second from left), the Harmony-Leiden pipeline (third from left), and scFormer (rightmost)

scFormer was first benchmarked against established batch-correction methods, including Harmony, BBKNN, MNN Correct, and ComBat. UMAP visualizations revealed pronounced batch effects prior to correction, manifesting as distribution shifts among samples. After correction, batch mixing was substantially improved by scFormer and Harmony, whereas weaker correction was observed for the remaining baselines in this dataset (Fig. 4a). Quantitative metrics further supported improved batch effect mitigation by scFormer, with a higher iLISI score (2.695 vs. 2.191 for Harmony) and a higher batch entropy (1.085 vs. 0.842 for Harmony) (Fig. 4b and Supplementary Fig. 3a).

We then examined how integration influenced rare-cell recovery. Rare-cell detection methods generally do not account for batch effects, and batch-associated structure can therefore be mistaken for biological rarity in multi-sample analyses. Conversely, workflows that first correct batch effects and then cluster cells may attenuate subtle transcriptional differences that are essential for identifying rare populations.

Based on these considerations, scFormer was compared with two alternatives: (i) scCAD applied in its standard configuration without additional batch effect correction, and (ii) a two-stage pipeline in which Harmony was first used for batch effect correction, followed by Leiden clustering, with clusters comprising ≤ 5% of cells designated as rare. Under this predefined Harmony-Leiden setting, using rare populations defined from the original study annotations as reference labels, scFormer achieved an F1 score of 0.675 (precision 0.977; recall 0.516), higher than both the Harmony-Leiden pipeline (F1 0.510) and scCAD (F1 0.190) (Fig. 4c). To make the parameter dependence of this two-stage baseline explicit, we additionally evaluated Harmony-Leiden across Leiden resolutions from 0.2 to 3.0, with the complete results provided in Supplementary Table 2.

To further dissect the contribution of the batch-correction loss, we performed an ablation analysis in which the two components described in Methods were evaluated separately: the batch-level distribution-alignment component and the nearest-neighbor local-structure component. Rare-cell F1 remained stable in the no-batch, distribution-component-only, and local-structure-component-only settings (0.663, 0.662, and 0.663, respectively), whereas the full model achieved the highest F1 score (0.675). In contrast, batch-mixing metrics improved substantially after adding batch-correction components: iLISI increased from 1.176 in the no-batch setting to 2.120, 2.445, and 2.695 in the distribution-component-only, local-structure-component-only, and full-model settings, respectively; batch entropy increased from 0.167 to 0.819, 0.953, and 1.085, respectively. These results indicate that the batch-correction loss improved batch mixing without reducing rare-cell recovery in this PBMC benchmark, and that the local-structure component made an important contribution to integration performance (Supplementary Fig. 4).

Closer examination suggests two distinct failure modes in the competing methods. scCAD, which lacks explicit batch effect correction, was sensitive to batch-associated variation and classified plasmacytoid dendritic cells (pDCs) as a rare cluster under the predicted-cluster abundance criterion (cluster 46), even though pDCs did not meet the rarity criterion in this dataset (Supplementary Fig. 3b). This pattern is consistent with technical variation being confounded with biological rarity when integration is omitted. In contrast, although the Harmony-Leiden workflow effectively reduced batch effects, the sequential “correct-then-cluster” strategy obscured biologically meaningful structure: Harmony merged hematopoietic stem cells and transitional B cells with lymphoid progenitors into a single artifactual cluster (Fig. 4d) and assigned an abundant CD4 + T cell population (17.43%) to a predicted rare cluster under the evaluation criterion (Supplementary Fig. 3b). The resolution sweep further showed that Harmony-Leiden results varied with the Leiden resolution parameter: lower resolutions produced fewer predicted rare cells with higher precision but lower recall, whereas higher resolutions increased the number of predicted rare-cell calls and reduced precision. These results provide a transparent assessment of the parameter sensitivity of the Harmony-Leiden baseline (Supplementary Table 2).

By jointly optimizing batch correction and rare-cell detection within a single framework, scFormer resolved five biologically plausible rare populations: B1 B cells (1.85%), proerythroblasts (1.42%), transitional B cells (0.89%), lymphoid progenitors (1.46%), and plasma cells (1.51%) (Fig. 4d). Together, these results indicate that an integrated approach mitigates false positives arising from batch effects while preserving fine-grained biological heterogeneity that may be diminished by sequential pipelines.

### scFormer reveals obscured rare epithelial states in the mouse airway

To further evaluate rare-cell detection performance, we applied scFormer and the next-best comparator, scCAD, to a single-cell RNA-sequencing dataset of mouse airway epithelial cells (Montoro et al. 2018). The dataset comprises 7,193 cells and 16,401 detected genes, with the original study annotating seven cell types, including two established rare populations: *Foxi1* + lung ionocytes and goblet cells (Supplementary Fig. 5a).

**Fig. 5:**
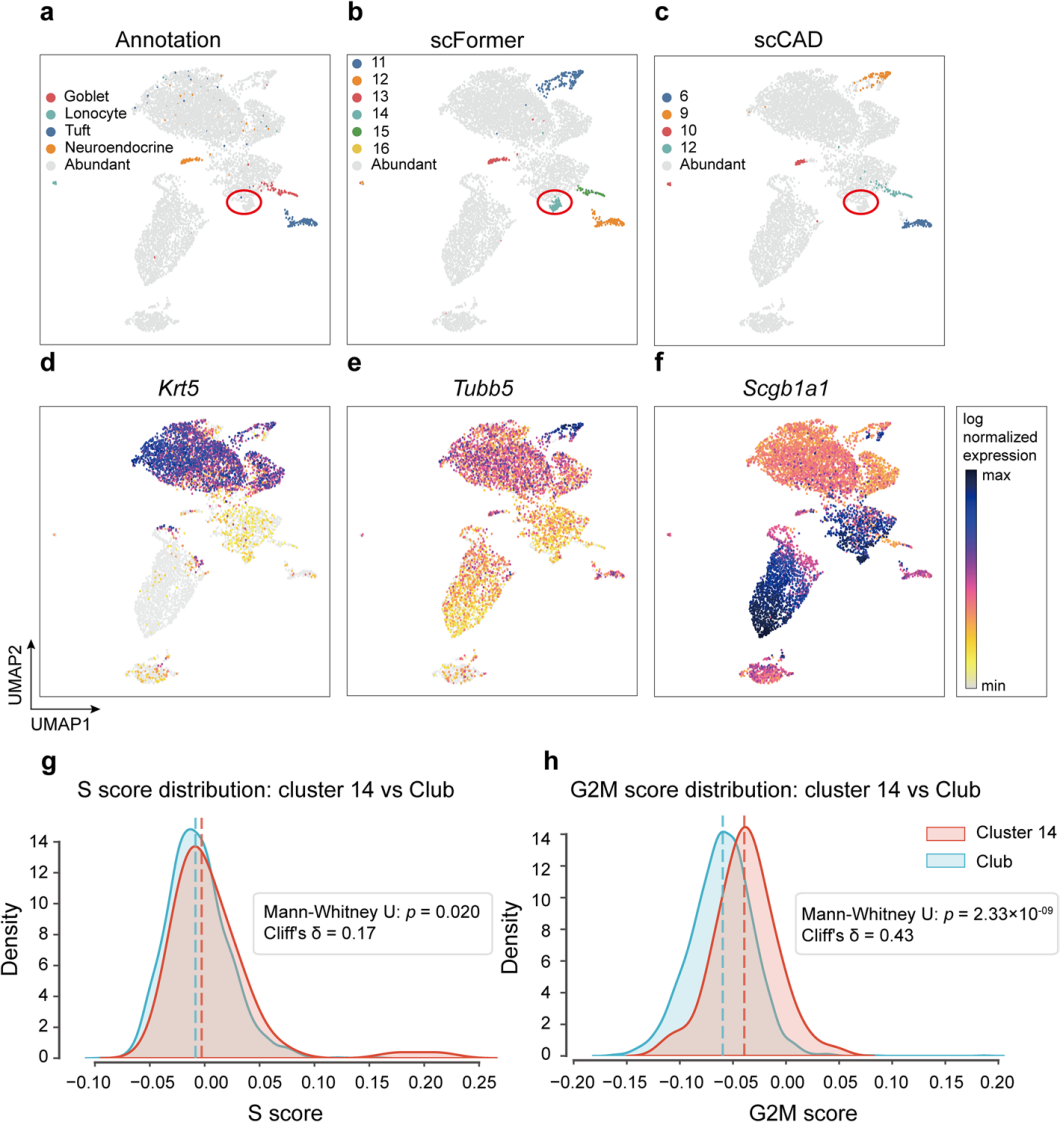
scFormer identifies known rare populations and candidate proliferative epithelial rare states in the mouse airway epithelium. **a-c** UMAP projections highlighting the distribution of rare-cell clusters (clusters comprising < 5% of cells), as defined by the original annotations (**a**), scFormer (**b**), and scCAD (**c**). Cluster 14, which is uniquely detected by scFormer, is indicated by a red circle. **d-f** UMAP feature plots showing normalized expression of key marker genes: the basal cell marker *Krt5* (**d**), the proliferation-associated gene *Tubb5* (**e**), and the club cell marker *Scgb1a1* (**f**). The color scale in (**d-f**) represents the level of gene expression, from low (gray) to high (midnight blue). **g**, **h** Density plots comparing cell-cycle scores between the scFormer-identified club-cell-associated rare cluster (cluster 14, red) and the remaining club cells (blue). **g** Distribution of S phase scores, showing modest right shift for cluster 14 (median = 0.00 vs. −0.01; *p* = 2.00 × 10⁻^2^, two-sided Mann–Whitney U test; Cliff’s δ = 0.17). **h** Distribution of G2/M phase scores, showing a pronounced right shift for cluster 14 (median = −0.04 vs. −0.06; *p* = 2.33 × 10^−^⁹, two-sided Mann–Whitney U test; Cliff’s δ = 0.43). Shaded areas denote the region of overlap between distributions; dashed lines indicate group medians

scFormer resolved 17 clusters (Supplementary Fig. 5b), six of which had abundance ≤ 5% (clusters 11–16; Fig. 5b). Cluster identities were assigned by computing Jaccard similarity between scFormer-defined clusters and annotated cell types, using marker genes reported in the original study together with the top 50 markers per cluster. Clusters 12, 13, 15 and 16 showed the highest similarity to tuft, neuroendocrine, goblet and *Foxi1* + ionocyte annotations, respectively (Supplementary Fig. 5c; Supplementary Table 4). Consistent marker expression further supported the recovery of rare populations reported in the original study.

Notably, both scFormer and scCAD identified cluster 11 as rare (Fig. 5a-c). However, the original annotations suggested that this cluster likely represents a heterogeneous admixture of basal and club cells. Jaccard analysis revealed minimal similarity to basal cells (J = 0.009) or tuft cells (J = 0.007), indicating that its biological identity resists simple classification (Supplementary Fig. 5c). Further analysis confirmed significant overexpression of basal cell markers (*Krt5*, *Trp63*) combined with specific upregulation of proliferation-associated genes (*Tubb5*, *Hmgb2*, *H2afv*) in cluster 11 (Fig. 5d-e and Supplementary Dataset 3). Given the involvement of these genes in cell-cycle control, DNA replication and mitosis (Hewitt et al. 2023; Rock et al. 2009; Saillour et al. 2014; Jiang et al. 2024; Tang et al. 2020), cluster 11 may represent a basal-cell-associated rare transcriptional state with proliferative features, rather than a fully resolved rare basal-cell type. Thus, scFormer not only recovered clusters concordant with manually annotated rare populations but also highlighted candidate rare states associated with distinct biological programs, including active proliferation.

Rare clusters are often characterized by both low abundance and weak transcriptional signatures that are easily masked by dominant populations. In this dataset, scFormer detected cluster 14 (0.92%) within the club-cell lineage, which was not identified by scCAD (Fig. 5b-c). Its club-cell identity was supported by the highest Jaccard similarity to annotated club cells and concordant expression of canonical markers such as *Scgb1a1* (Martinu et al. 2023) (Fig. 5f, Supplementary Dataset 3). To further characterize this population, we performed cell-cycle profiling using a murine gene set (v2021) from the GitHub repository hbc/tinyatlas. Cluster 14 exhibited significantly elevated cell-cycle activity relative to other club cells (Mann–Whitney U test, two-sided; S phase: *p* = 2.00 × 10^–2^, Cliff’s δ = 0.17; G2/M phase: *p* = 2.33 × 10^–9^, Cliff’s δ = 0.43), consistent with proliferative features (Fig. 5g-h). Whereas increases in S-phase activity were modest, the pronounced G2/M shift suggested enrichment of G2/M-associated transcriptional programs in this cluster. These results support cluster 14 as a rare club-cell-associated transcriptional state with proliferative features, while motivating additional controls to assess whether its recovery was driven primarily by cell-cycle-associated genes.

To further examine whether these rare airway clusters were driven primarily by cell-cycle-associated genes, we reran scFormer after removing 94 canonical murine S/G2M genes detected in this dataset, while keeping the same preprocessing, clustering, and model parameters. The club-cell-associated cluster remained detectable after this control: its best-matched cluster contained 44 cells, all of which originated from the original cluster 14 (Jaccard = 0.667). These cells retained uniform expression of club-cell markers, including *Scgb1a1*, *Scgb3a1*, *Scgb3a2*, and *Cyp2f2*, and showed no detectable *Mki67* or *Top2a* expression. The basal-cell-associated cluster also retained a high-purity matched cluster after cell-cycle gene removal, with 84 of 87 cells originating from the original cluster 11 (Jaccard = 0.522), and retained expression of basal markers including *Krt5* and *Trp63*. These results indicate that the rare airway clusters were not solely attributable to S/G2M gene-driven clustering, although the partial recovery of cluster 11 supports a more conservative interpretation of this population as a basal-cell-associated rare transcriptional state with proliferative features (Supplementary Fig. 6).

### scFormer recovers revival stem cells and resolves injury-associated rare immune subsets in mouse intestinal crypts

To assess whether scFormer can identify rare populations without manual guidance, we analyzed a mouse intestinal scRNA-seq dataset comprising 6,644 single-cell transcriptomes from intestinal crypts collected during regeneration after epithelial injury. Intestinal homeostasis depends on *LGR5* + crypt-base columnar cells (CBCs), which are depleted following damage and transiently replaced by rare, clusterin (*Clu*)-expressing revival stem cells (revSCs) that drive regeneration (Ayyaz et al. 2019; Barker et al. 2007; Metcalfe et al. 2014; Tao et al. 2015).

**Fig. 6:**
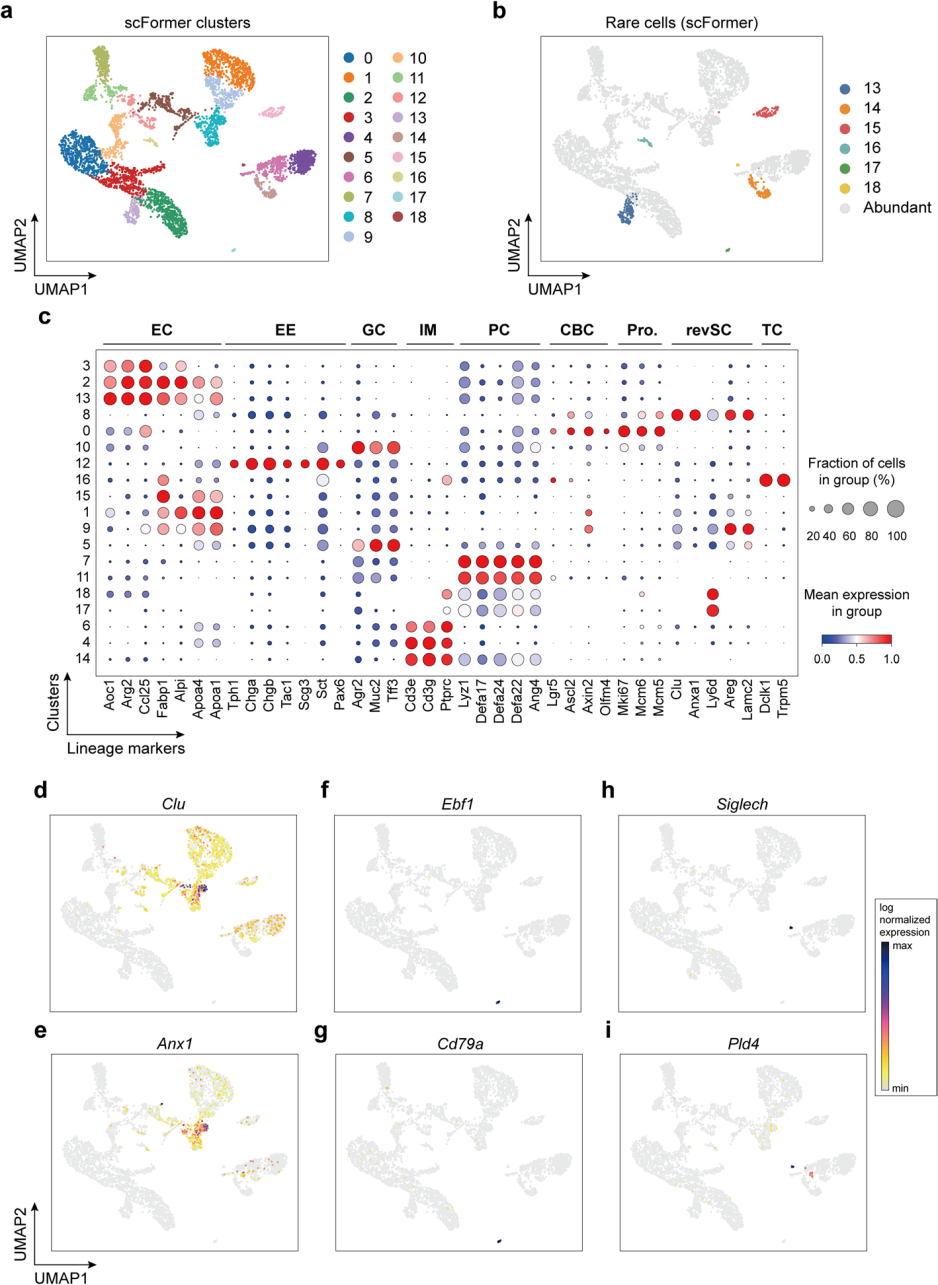
scFormer uncovers rare-cell clusters in unannotated mouse intestinal epithelium. **a** UMAP projection of 6,644 mouse intestinal epithelium cells, colored by the 19 clusters identified by scFormer. **b** The same UMAP embedding as in (**a**), with the six rare clusters highlighted. **c** Dot plot showing the expression of canonical lineage markers used for cell-type annotation. Dot size denotes the percentage of cells within each cluster expressing a given gene, and color intensity reflects mean expression (from low to high). Cell populations are annotated as: Enterocytes (EC), Enteroendocrine cells (EE), Goblet cells (GC), Paneth cells (PC), Tuft cells (TC), Crypt Base Columnar cells (CBC), reviving Stem Cells (revSC), Proliferating cells (Pro.), and Immune cells (IM). **d-i** UMAP feature plots displaying the normalized expression of representative markers: *Clu*, *Anxa1*, *Ebf1*, *Cd79a*, *Siglech* and *Pld4*. The color scale in (**d-i**) represents the level of gene expression, from low (gray) to high (midnight blue)

scFormer partitioned the dataset into 19 clusters and recovered six rare populations (≤ 5% frequency), three of which represented < 1% of all cells (Fig. 6a-b). Comparison with the major lineages reported in the original study revealed overall concordance (Fig. 6c and Supplementary Table 5). Clusters 13 and 15 mapped to enterocytes (Jaccard similarity > 0.34), cluster 16 mapped to tuft cells (Jaccard = 0.40), and cluster 14 mapped to the dominant lymphocyte population (Jaccard = 0.27) (Fig. 6c and Supplementary Table 5). Notably, revSCs were recovered as cluster 8 (Fig. 6c; Supplementary Table 5), which was enriched in irradiated samples and exhibited strong expression of canonical revSC markers, including *Clu* and *Anxa1* (Fig. 6d-e; Supplementary Dataset 4). These results indicate that scFormer reconstructs established intestinal architecture and recovers a rare regenerative population without relying on predefined labels.

Beyond previously reported populations, scFormer further resolved two low-frequency immune clusters that were not emphasized in the original analysis. Both were detected almost exclusively in non-irradiated samples and were markedly diminished after injury (Supplementary Fig. 7a-b), suggesting state-dependent immune composition at homeostasis. Although broadly classified as lymphocytes, their Jaccard similarity to the main lymphocyte population was extremely low (< 0.05), indicating distinct, previously uncharacterized subtypes (Supplementary Table 5).

The first subset, Cluster 17, represented a B-cell subpopulation expressing *Ebf1*, *Cd79a*, and MHC-II molecules (*H2-Aa*, *H2-Ab1*) (Fig. 6f-g), consistent with a role in immune surveillance and tolerance to commensal microbiota, with its loss after injury potentially reflecting radiation sensitivity or inflammatory redistribution (Fedl et al. 2024; Davis et al. 2010; Chen et al. 2018; Suzuki et al. 2023). The second subset, Cluster 18 was identified as a plasmacytoid dendritic cell (pDC) subset marked by *Siglech*, *Pld4*, and *Irf8* (Fig. 6h-i). Given their capacity for type I interferon production, their disappearance during injury may help prevent excessive inflammation that could hinder epithelial repair (Ascic et al. 2024; Gavin et al. 2021; Valente et al. 2023). Collectively, these findings highlight scFormer’s ability to uncover functionally distinct rare subsets and provide new insights into the immune regulation of the intestinal niche.

### scFormer enables rare-cell identification in spatial transcriptomics

**Fig. 7:**
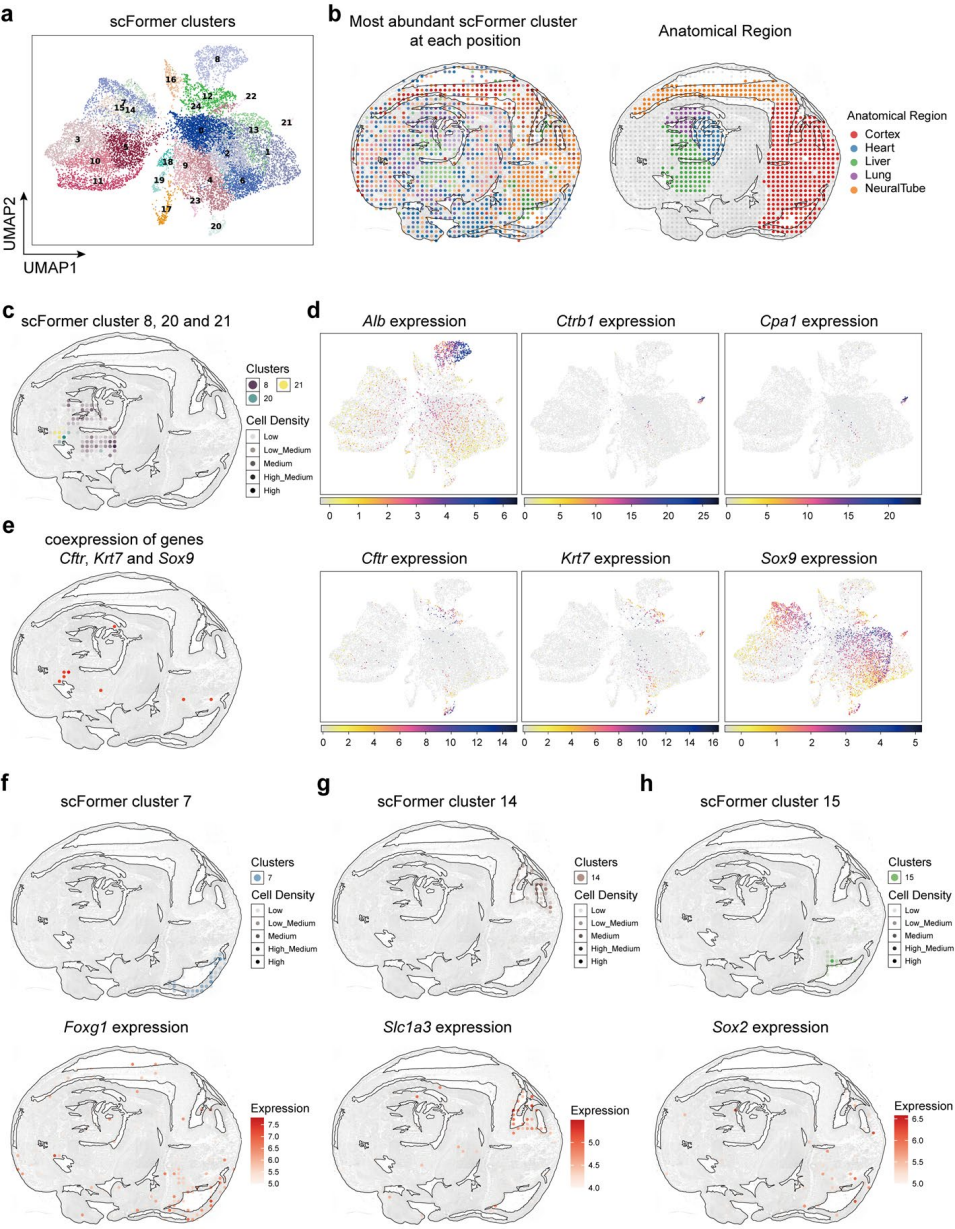
scFormer identifies rare cell states in spatial transcriptomic data. **a** UMAP projection of 16,222 cells from an E14 mouse embryonic sagittal section (slide_4E), colored by the 25 clusters identified by scFormer. Slide_4E is shown because it yields the highest recovery of nuclei. **b** Left, spatial map of the embryonic section, with each spot colored by its dominant cluster as determined by scFormer. Right, corresponding anatomical annotations of major structures, including cortex, heart, liver, lung, and neural tube. **c** Spatial distribution of clusters 8, 20 and 21. These clusters show spatially organized distributions consistent with hepatic and pancreatic territories. Color intensity reflects local cell density. **d** UMAP feature plots showing expression patterns of representative marker genes. The top row displays the hepatocyte marker *Alb* and pancreatic exocrine markers *Ctrb1* and *Cpa1*. The bottom row displays biliary epithelial markers *Cftr*, *Krt7* and *Sox9*. Color intensity reflects expression level from low (gray) to high (midnight blue). **e** Spatial visualization of cells co-expressing *Cftr*, *Krt7* and *Sox9* (red), showing the anatomical distribution of the biliary epithelial-marker-positive subset. **f–h** Spatial distribution (top) and corresponding marker expression (bottom) for three neurodevelopmental populations: cluster 7 (forebrain lineage, *Foxg1* +) (**f**), cluster 14 (radial glia, *Slc1a3* +) (**g**) and cluster 15 (neural stem/progenitor cells, *Sox2* +) (**h**)

We next evaluated scFormer in a spatial transcriptomics setting using the sci-Space dataset, which comprises 52,636 spatially resolved gene-expression profiles derived from 122,278 single-cell transcriptomes from E14 mouse embryonic sagittal sections (Srivatsan et al. 2021). Using gene expression profiles as input, scFormer identified 29 transcriptionally distinct populations (Supplementary Fig. 8a). For detailed evaluation, we focused on a representative tissue section, slide_4E (16,222 cells). Within this section, scFormer identified 25 clusters (Fig. 7a), and these clusters were then examined in their original spatial coordinates. The spatial distribution of these clusters was broadly consistent with the reference anatomical annotations (Fig. 7b). Specifically, scFormer recovered anatomically widespread stromal populations, such as fibroblasts (*Fstl1* +, Supplementary Fig. 9a), while also distinguishing organ-associated parenchymal populations, including cardiac (*Myh7* +) (Gacita et al. 2021), hepatic (*Alb* +) (Hung et al. 2022), and spinal cord (*Plxna2* +) (Sekine et al. 2019) clusters, each showing localization consistent with the expected anatomical domains (Supplementary Fig. 9b-d). To quantitatively evaluate spatial organization beyond visual inspection, we performed three post hoc analyses using the original tissue coordinates and published sci-Space annotations: k-nearest-neighbor spatial self-enrichment, hypergeometric enrichment against published annotations, and marker/module expression enrichment. These analyses are summarized in Supplementary Fig. 11 and Supplementary Datasets 5–7.

These analyses further show that scFormer can identify rare cell states in spatial transcriptomic data and allow their anatomical distributions to be examined after mapping cluster labels back to the original tissue coordinates. In the hepatic region of slide_4E, scFormer distinguished the abundant hepatocyte population (Cluster 8, Alb +) from a rare biliary epithelial-like cell state (Cluster 20; 0.92%, n = 149). After projection back to the original tissue coordinates, Cluster 20 showed significant spatial self-enrichment relative to the permutation background (0.326 observed versus 0.009 permutation background; 35.74-fold enrichment; BH-adjusted empirical *q* = 1.14 × 10^−4^) and showed a distribution consistent with developing biliary structures (Fig. 7c). Consistent with its hepatic location, Cluster 20 was enriched for the published Liver anatomical annotation (57/149 cells; 6.02-fold enrichment; BH-adjusted *q* = 1.94 × 10^−29^). A subset of cells in Cluster 20 co-expressed *Cftr*, *Krt7*, and *Sox9* (14.8% of Cluster 20 cells; 4.15-fold higher mean module expression than other cells), a multigene signature characteristic of biliary epithelium (Amarachintha et al. 2022; Liu et al. 2022; Rimland et al. 2021), supporting its annotation as a biliary epithelial-like cell state. Spatial visualization of cells co-expressing these markers further showed that this marker-positive subset was concentrated within the hepatic region (Fig. 7e). Detailed spatial enrichment, annotation enrichment, and marker-module statistics for Cluster 20 are summarized in Supplementary Fig. 11 and Supplementary Datasets 5–7. These findings indicate that rare cell states identified by scFormer from spatial transcriptomic expression profiles can be examined in their original anatomical context.

scFormer also identified an additional rare cell state. Cluster 21 represented a rare subset (0.52%, n = 84) without the *Cftr*/*Krt7*/*Sox9* co-expression pattern used for Cluster 20 but exhibiting enrichment of pancreatic marker genes *Ctrb1*, *Cpa1*, and *Clps* (Bodas et al. 2025; Tamura et al. 2018; Zhang et al. 2013), as shown in the UMAP feature plots and supplementary marker visualizations (Fig. 7d and Supplementary Fig. 10a-d). Spatial mapping showed that these cells could be examined in their original tissue coordinates, with a distribution shown in Fig. 7c. Cluster 21 also showed strong spatial self-enrichment relative to the permutation background (0.461 observed versus 0.005 permutation background; 89.52-fold enrichment; BH-adjusted empirical *q* = 1.14 × 10^−4^). Annotation-level comparison with published sci-Space labels showed that Cluster 21 was enriched in pancreatic-associated annotation categories, including Pancreatic Islet Cells in manual_annotation_2 (61/84 cells; 193.12-fold enrichment; BH-adjusted *q* = 2.36 × 10^−152^) and Epithelial Cells in final_cluster_label (61/84 cells; 9.42-fold enrichment; BH-adjusted *q* = 9.62 × 10^−49^). Consistent with the marker visualizations, Ctrb1/Cpa1 module expression was enriched in Cluster 21, with 56.0% of Cluster 21 cells co-expressing both markers. Detailed spatial enrichment, annotation enrichment, and marker-module statistics for Cluster 21 are summarized in Supplementary Fig. 11 and Supplementary Datasets 5–7. These results support the description of Cluster 21 as a pancreatic marker-enriched rare cell state that can be examined within the original tissue coordinates.

scFormer further delineated rare neurodevelopmental populations. Cluster 7 (5.31%, n = 862) showed high *Foxg1* expression, consistent with a telencephalic lineage, and its spatial distribution overlapped forebrain cortical regions (Fig. 7f) (Mariani et al. 2015). Cluster 14 (2.82%, n = 458) expressed the glial marker *Slc1a3* together with Notch-pathway components such as *Hes5*, mapping to the ventricular zone characteristic of radial glia (Fig. 7g) (Bifari et al. 2017; Ruan et al. 2021). Cluster 15 (2.22%, n = 360) showed high *Sox2* expression, consistent with neural stem and progenitor identity (Fig. 7h) (Jung et al. 2020). Consistent with these visual patterns, clusters 7, 14, and 15 showed 13.25- to 22.57-fold spatial self-enrichment over permutation background, and their published annotation and marker-enrichment results are summarized in Supplementary Fig. 11 and Supplementary Datasets 5–7. Collectively, these results support the use of scFormer for identifying rare populations in spatial transcriptomic data and examining their anatomical distributions within embryonic tissue sections.

## Discussion

In this study, we introduce scFormer, a HGT-based computational framework designed to address the dual challenges of identifying rare cells in scRNA-seq data while simultaneously mitigating batch effects in multi-sample analyses. Through a specificity-driven cell-gene heterogeneous graph and an optional batch-correction module, scFormer achieves high accuracy, robustness and practical reliability in rare-cell detection.

scFormer was rigorously evaluated across 125 simulated and 18 real scRNA-seq datasets, where it consistently outperformed existing approaches. In simulations, scFormer achieved the highest F1 score (0.988) and maintained stable performance across both high- and low-separation scenarios. By contrast, methods such as RaceID3, FiRE, and GiniClust3 exhibited substantial performance degradation under low-separation conditions, reflecting limited ability to detect rare cells with weak transcriptional signatures.

In real datasets, scFormer achieved a mean F1 score of 0.595, representing a 22.1% and 93.8% improvement over scCAD (0.488) and GiniClust3 (0.307), respectively. For example, in a mouse retina dataset, scFormer reached an F1 score of 0.955, markedly exceeding scCAD (0.649) and GiniClust3 (0.282). These gains are consistent with the model design. First, the construction of specificity-driven cell-gene heterogeneous graphs captures rare transcriptional programs that are often under-represented in cell–cell graphs that rely primarily on homophily. Second, the heterogeneous graph transformer architecture explicitly models distinct biological relationships, preserving subtle rare-cell signals within high-dimensional data. Mechanistically, this provides a direct rationale for why rare clusters can still be resolved when their same-state cellular neighbors are limited.

Recent transformer-based graph frameworks have highlighted that joint analysis of RNA- and ATAC-seq data can improve the resolution of rare cell states in cancer, as exemplified by MarsGT (Wang et al. 2024). In a matched lymphoma dataset from 10 × Genomics, MarsGT reported that B-cell subpopulations could not be effectively distinguished when using RNA-only or ATAC-only data, whereas joint modeling recovered a rare B-lymphoma precursor-like state (BLS1) within B cells. Notably, scFormer recovered an analogous rare B-cell state using the RNA-seq data alone (cluster 13; Supplementary Fig. 12), suggesting that a specificity-driven cell-gene topology can amplify weak transcriptomic signatures even without additional modality. Given the technical challenges and higher costs associated with acquiring paired RNA-seq and ATAC-seq data, scFormer demonstrates broader applicability as an RNA-first framework. It serves not merely as a baseline, but as a robust foundation for future multi-omics extensions.

The practical value of scFormer is further enhanced by its batch-correction capability. By combining global distribution alignment with local structure preservation, scFormer mitigates batch effects while retaining rare-cell signatures. This design avoids relying on batch-level distribution alignment alone: rare-cell information is first represented through the specificity-driven cell-gene graph, while the local-structure component constrains the embedding so that fine-grained neighborhood relationships are maintained during integration. In a PBMC dataset, scFormer achieved an iLISI score of 2.695, outperforming Harmony (2.191), demonstrating effective removal of technical artifacts without obscuring biologically relevant variation. The component ablation further showed that separating the distribution-alignment and local-structure components maintained rare-cell F1 while improving batch mixing, and that the full model achieved the strongest overall integration metrics. These results support the view that local structure preservation helps balance batch alignment with rare-cell recovery.

The wide applicability of scFormer was further validated across diverse biological contexts. In the mouse airway epithelial cell dataset, scFormer recovered known rare types, including *Foxi1* + lung ionocytes and goblet cells, while highlighting basal-cell-associated (cluster 11) and club-cell-associated (cluster 14) rare transcriptional states with proliferative features. These rare airway clusters were further supported by the S/G2M gene-removal control, indicating that they were not solely attributable to cell-cycle-gene-driven clustering. In a large-scale human T-cell dataset, scFormer detected proliferating cells, plasmacytoid dendritic cells (pDCs), platelets, and hematopoietic stem and progenitor cells (HSPCs), and further identified a monocyte-derived macrophage subpopulation with M2-like polarization characteristics (cluster 19). In the unannotated mouse intestinal epithelium dataset, scFormer recovered revival stem cells (revSCs), and uncovered two rare lymphocyte subpopulations (B cells and pDCs) absent from the original analysis. These subpopulations, exclusive to non-irradiated samples, suggest specialized roles in immune homeostasis, illustrating the potential of scFormer for discovering functionally relevant rare cells without prior annotations. In spatial transcriptomics, scFormer identified 29 clusters, several of which showed anatomical consistency when examined in the original tissue coordinates. This spatial organization was further supported by k-nearest-neighbor spatial self-enrichment analysis, enrichment against published sci-Space annotations, and marker/module expression quantification, including support for biliary epithelial-like and pancreatic marker-enriched rare cell states. Together, these results indicate that the scFormer formulation is not restricted to a single experimental setting and can be applied to both conventional scRNA-seq and spatially resolved transcriptomic profiles.

A further indicator of robustness is that scFormer successfully executed on all 18 real datasets (100% success rate), whereas every competing method failed on at least one dataset (Supplementary Dataset 2). This reliability underscores its stability and generalizability for rare-cell identification. Nevertheless, several directions remain. First, although scFormer is designed as an RNA-first framework, extending the specificity-driven graph to incorporate additional modalities or emerging biomolecular detection technologies, such as chromatin accessibility and nanopore-based biomolecule detection, may enhance rare-state resolution and enable more mechanistic interpretation (Li et al. 2024), while transcriptome-scale perturbation strategies such as CRISPR-Cas13 screening may help functionally validate candidate rare-cell programs (Si et al. 2025). Second, although the K-sensitivity analysis supports *K* = 20 as a practical default in simulated rare-cell benchmarks, broader evaluation of graph sparsity and other hyperparameters across additional tissues, sequencing depths and spatial platforms, as well as future adaptive gene-selection strategies based on dataset-specific specificity thresholds, may further refine dataset-specific defaults for routine use.

In conclusion, the performance of scFormer derives from its specificity-aware graph architecture and integrated batch-correction strategy, which together address central limitations of existing approaches. By prioritizing biologically meaningful signals while controlling technical noise, scFormer provides a reliable and versatile framework for discovery in single-cell datasets.

## Supplementary Information

Supplementary Material 1.

Supplementary Material 2.

## Data Availability

Details of the datasets used in this manuscript are provided in Supplementary Table 3. All described datasets were obtained from various public websites using the accession codes provided in Supplementary Table 3, including the NCBI Gene Expression Omnibus (GEO) [https://www.ncbi.nlm.nih.gov/geo/]. The 10 × PBMC dataset is obtained from GitHub [https://github.com/ttgump/scDeepCluster/blob/master/scRNA-seq%20data/10X_PBMC.h5]. The preprocessed human tonsil data are available from the Broad Institute Single Cell Portal [https://singlecell.broadinstitute.org/single_cell/study/SCP2169/slide-tags-snrna-seq-onhuman-tonsil]. scFormer is publicly available at GitHub [https://github.com/StickTaTa/ScFormer].

## References

[CR1] Amarachintha SP, Mourya R, Ayabe H, Yang L, Luo Z, Li X, et al. Biliary organoids uncover delayed epithelial development and barrier function in biliary atresia. Hepatology. 2022;75(1):89–103. 10.1002/hep.32107. 34392560 10.1002/hep.32107 PMC9983428

[CR2] Argelaguet R, Cuomo ASE, Stegle O, Marioni JC. Computational principles and challenges in single-cell data integration. Nat Biotechnol. 2021;39(10):1202–15. 10.1038/s41587-021-00895-7. 33941931 10.1038/s41587-021-00895-7

[CR3] Ascic E, Åkerström F, Sreekumar Nair M, Rosa A, Kurochkin I, Zimmermannova O, et al. In vivo dendritic cell reprogramming for cancer immunotherapy. Science. 2024;386(6719):eadn9083. 10.1126/science.adn9083. 39236156 10.1126/science.adn9083 PMC7616765

[CR4] Ayyaz A, Kumar S, Sangiorgi B, Ghoshal B, Gosio J, Ouladan S, et al. Single-cell transcriptomes of the regenerating intestine reveal a revival stem cell. Nature. 2019;569(7754):121–5. 10.1038/s41586-019-1154-y. 31019301 10.1038/s41586-019-1154-y

[CR5] Barker N, van Es JH, Kuipers J, Kujala P, van den Born M, Cozijnsen M, et al. Identification of stem cells in small intestine and colon by marker gene Lgr5. Nature. 2007;449(7165):1003–7. 10.1038/nature06196. 17934449 10.1038/nature06196

[CR6] Belarif L, Vanhove B, Poirier N. Full antagonist of the IL-7 receptor suppresses chronic inflammation in non-human primate models by controlling antigen-specific memory T cells. Cell Stress. 2018;2(12):362–4. 10.15698/cst2018.12.168. 31225460 10.15698/cst2018.12.168 PMC6551675

[CR7] Bifari F, Decimo I, Pino A, Llorens-Bobadilla E, Zhao S, Lange C, et al. Neurogenic radial glia-like cells in meninges migrate and differentiate into functionally integrated neurons in the neonatal cortex. Cell Stem Cell. 2017;20(3):360-373.e7. 10.1016/j.stem.2016.10.020. 27889318 10.1016/j.stem.2016.10.020

[CR8] Bodas C, Felipe I, Chanez B, Lafarga M, Lopez de Maturana E, Martinez de Villarreal J, et al. A common CTRB misfolding variant associated with pancreatic cancer risk causes ER stress and inflammation in mice. Gut. 2025. 10.1136/gutjnl-2024-333406. 40254337 10.1136/gutjnl-2024-333406

[CR9] Chen Y-T, Su Y-C, Kung JT. B cell development sans B cell receptor responsiveness due to unfolded protein response-triggered Mef2c protein degradation. J Immunol Baltim Md 1950. 2018;201(10):2885–98. 10.4049/jimmunol.1800685. 10.4049/jimmunol.1800685 30305329

[CR10] Choi YH, Kim JK. Dissecting cellular heterogeneity using single-cell RNA sequencing. Mol Cells. 2019;42(3):189–99. 10.14348/molcells.2019.2446. 10.14348/molcells.2019.2446 PMC6449718 30764602

[CR11] Chu X, Tian W, Ning J, Xiao G, Zhou Y, Wang Z, et al. Cancer stem cells: advances in knowledge and implications for cancer therapy. Signal Transduct Target Ther. 2024;9(1):170. 10.1038/s41392-024-01851-y. 38965243 10.1038/s41392-024-01851-y PMC11224386

[CR12] Davis RE, Ngo VN, Lenz G, Tolar P, Young RM, Romesser PB, et al. Chronic active B-cell-receptor signalling in diffuse large B-cell lymphoma. Nature. 2010;463(7277):88–92. 10.1038/nature08638. 20054396 10.1038/nature08638 PMC2845535

[CR13] Deng Q, Ramsköld D, Reinius B, Sandberg R. Single-cell RNA-seq reveals dynamic, random monoallelic gene expression in mammalian cells. Science. 2014;343(6167):193–6. 10.1126/science.1245316. 24408435 10.1126/science.1245316

[CR14] Dong R, Yuan G-C. GiniClust3: a fast and memory-efficient tool for rare cell type identification. BMC Bioinformatics. 2020;21(1):158. 10.1186/s12859-020-3482-1. 32334526 10.1186/s12859-020-3482-1 PMC7183612

[CR15] Dou J, Liang S, Mohanty V, Miao Q, Huang Y, Liang Q, et al. Bi-order multimodal integration of single-cell data. Genome Biol. 2022;23(1):112. 10.1186/s13059-022-02679-x. 35534898 10.1186/s13059-022-02679-x PMC9082907

[CR16] Fa B, Wei T, Zhou Y, Johnston L, Yuan X, Ma Y, et al. GapClust is a light-weight approach distinguishing rare cells from voluminous single cell expression profiles. Nat Commun. 2021;12(1):4197. 10.1038/s41467-021-24489-8. 34234139 10.1038/s41467-021-24489-8 PMC8263561

[CR17] Fedl AS, Tagoh H, Gruenbacher S, Sun Q, Schenk RL, Froussios K, et al. Transcriptional function of E2A, Ebf1, Pax5, Ikaros and Aiolos analyzed by in vivo acute protein degradation in early B cell development. Nat Immunol. 2024;25(9):1663–77. 10.1038/s41590-024-01933-7. 39179932 10.1038/s41590-024-01933-7

[CR18] Gacita AM, Fullenkamp DE, Ohiri J, Pottinger T, Puckelwartz MJ, Nobrega MA, et al. Genetic variation in enhancers modifies cardiomyopathy gene expression and progression. Circulation. 2021;143(13):1302–16. 10.1161/CIRCULATIONAHA.120.050432. 33478249 10.1161/CIRCULATIONAHA.120.050432 PMC8009836

[CR19] Galassi C, Chan TA, Vitale I, Galluzzi L. The hallmarks of cancer immune evasion. Cancer Cell. 2024;42(11):1825–63. 10.1016/j.ccell.2024.09.010. 39393356 10.1016/j.ccell.2024.09.010

[CR20] Gavin AL, Huang D, Blane TR, Thinnes TC, Murakami Y, Fukui R, et al. Cleavage of DNA and RNA by PLD3 and PLD4 limits autoinflammatory triggering by multiple sensors. Nat Commun. 2021;12(1):5874. 10.1038/s41467-021-26150-w. 34620855 10.1038/s41467-021-26150-w PMC8497607

[CR21] Goddard ET, Linde MH, Srivastava S, Klug G, Shabaneh TB, Iannone S, et al. Immune evasion of dormant disseminated tumor cells is due to their scarcity and can be overcome by T cell immunotherapies. Cancer Cell. 2024;42(1):119-134.e12. 10.1016/j.ccell.2023.12.011. 38194912 10.1016/j.ccell.2023.12.011 PMC10864018

[CR22] Haghverdi L, Lun ATL, Morgan MD, Marioni JC. Batch effects in single-cell RNA-sequencing data are corrected by matching mutual nearest neighbors. Nat Biotechnol. 2018;36(5):421–7. 10.1038/nbt.4091. 29608177 10.1038/nbt.4091 PMC6152897

[CR23] Hao Y, Hao S, Andersen-Nissen E, Mauck WM, Zheng S, Butler A, et al. Integrated analysis of multimodal single-cell data. Cell. 2021;184(13):3573-3587.e29. 10.1016/j.cell.2021.04.048. 34062119 10.1016/j.cell.2021.04.048 PMC8238499

[CR24] Hao Y, Stuart T, Kowalski MH, Choudhary S, Hoffman P, Hartman A, et al. Dictionary learning for integrative, multimodal and scalable single-cell analysis. Nat Biotechnol. 2024;42(2):293–304. 10.1038/s41587-023-01767-y. 37231261 10.1038/s41587-023-01767-y PMC10928517

[CR25] Herman J, Sagar, Grün D. FateID infers cell fate bias in multipotent progenitors from single-cell RNA-seq data. Nat Methods. 2018;15(5):379–86. 10.1038/nmeth.4662. 10.1038/nmeth.4662 29630061

[CR26] Hewitt RJ, Puttur F, Gaboriau DCA, Fercoq F, Fresquet M, Traves WJ, et al. Lung extracellular matrix modulates KRT5+ basal cell activity in pulmonary fibrosis. Nat Commun. 2023;14(1):6039. 10.1038/s41467-023-41621-y. 37758700 10.1038/s41467-023-41621-y PMC10533905

[CR27] Hu Z, Dai M, Chang Y, Hua X, Zhang N, Chen X, et al. Strategies for arterial graft optimization at the single-cell level. Nat Cardiovasc Res. 2024;3(5):541–57. 10.1038/s44161-024-00464-6. 39195932 10.1038/s44161-024-00464-6

[CR28] Hung C-T, Su T-H, Chen Y-T, Wu Y-F, Chen Y-T, Lin S-J, et al. Targeting ER protein TXNDC5 in hepatic stellate cell mitigates liver fibrosis by repressing non-canonical TGFβ signalling. Gut. 2022;71(9):1876–91. 10.1136/gutjnl-2021-325065. 34933915 10.1136/gutjnl-2021-325065

[CR29] Jaitin DA, Kenigsberg E, Keren-Shaul H, Elefant N, Paul F, Zaretsky I, et al. Massively parallel single-cell RNA-seq for marker-free decomposition of tissues into cell types. Science. 2014;343(6172):776–9. 10.1126/science.1247651. 24531970 10.1126/science.1247651 PMC4412462

[CR30] Jiang J, Shao X, Liu W, Wang M, Li Q, Wang M, et al. The mechano-chemical circuit in fibroblasts and dendritic cells drives basal cell proliferation in psoriasis. Cell Rep. 2024;43(7):114513. 10.1016/j.celrep.2024.114513. 39003736 10.1016/j.celrep.2024.114513

[CR31] Jindal A, Gupta P, Jayadeva, Sengupta D. Discovery of rare cells from voluminous single cell expression data. Nat Commun. 2018;9(1):4719. 10.1038/s41467-018-07234-6. 10.1038/s41467-018-07234-6 PMC6226447 30413715

[CR32] Jung S, Choe S, Woo H, Jeong H, An H-K, Moon H, et al. Autophagic death of neural stem cells mediates chronic stress-induced decline of adult hippocampal neurogenesis and cognitive deficits. Autophagy. 2020;16(3):512–30. 10.1080/15548627.2019.1630222. 31234698 10.1080/15548627.2019.1630222 PMC6999625

[CR33] Korsunsky I, Millard N, Fan J, Slowikowski K, Zhang F, Wei K, et al. Fast, sensitive and accurate integration of single-cell data with Harmony. Nat Methods. 2019;16(12):1289–96. 10.1038/s41592-019-0619-0. 31740819 10.1038/s41592-019-0619-0 PMC6884693

[CR34] Levine JH, Simonds EF, Bendall SC, Davis KL, Amir ED, Tadmor MD, et al. Data-driven phenotypic dissection of AML reveals progenitor-like cells that correlate with prognosis. Cell. 2015;162(1):184–97. 10.1016/j.cell.2015.05.047. 26095251 10.1016/j.cell.2015.05.047 PMC4508757

[CR35] Li P, Liang D, Yang E, Zeb M, Huang H, Sun H, et al. Bio-nanopore technology for biomolecules detection. Adv Biotechnol. 2024;2(4):45. 10.1007/s44307-024-00051-7. 10.1007/s44307-024-00051-7 PMC11740844 39883334

[CR36] Liang S, Mohanty V, Dou J, Miao Q, Huang Y, Müftüoğlu M, et al. Single-cell manifold-preserving feature selection for detecting rare cell populations. Nat Comput Sci. 2021;1(5):374–84. 10.1038/s43588-021-00070-7. 36969355 10.1038/s43588-021-00070-7 PMC10035340

[CR37] Liao S, Zhou X, Liu C, Liu C, Hao S, Luo H, et al. Stereo-cell: spatial enhanced-resolution single-cell sequencing with high-density DNA nanoball-patterned arrays. Science. 2025;389(6762):eadr0475. 10.1126/science.adr0475. 40839715 10.1126/science.adr0475

[CR38] Liu Y, Zhuo S, Zhou Y, Ma L, Sun Z, Wu X, et al. Yap-Sox9 signaling determines hepatocyte plasticity and lineage-specific hepatocarcinogenesis. J Hepatol. 2022;76(3):652–64. 10.1016/j.jhep.2021.11.010. 34793870 10.1016/j.jhep.2021.11.010 PMC8858854

[CR39] Liu J, Fan X, Gu C, Yang Y, Wu B, Chen G, et al. scHeteroNet: a heterophily-aware graph neural network for accurate cell type annotation and novel cell detection. Adv Sci Weinh Baden-Wurtt Ger. 2025;12(16):e2412095. 10.1002/advs.202412095. 10.1002/advs.202412095 PMC12021051 40042052

[CR40] Lu Z, Mo S, Xie D, Zhai X, Deng S, Zhou K, et al. Polyclonal-to-monoclonal transition in colorectal precancerous evolution. Nature. 2024;636(8041):233–40. 10.1038/s41586-024-08133-1. 39478225 10.1038/s41586-024-08133-1

[CR41] Ma A, Wang X, Li J, Wang C, Xiao T, Liu Y, et al. Single-cell biological network inference using a heterogeneous graph transformer. Nat Commun. 2023;14(1):964. 10.1038/s41467-023-36559-0. 36810839 10.1038/s41467-023-36559-0 PMC9944243

[CR42] Mariani J, Coppola G, Zhang P, Abyzov A, Provini L, Tomasini L, et al. FOXG1-dependent dysregulation of GABA/glutamate neuron differentiation in autism spectrum disorders. Cell. 2015;162(2):375–90. 10.1016/j.cell.2015.06.034. 26186191 10.1016/j.cell.2015.06.034 PMC4519016

[CR43] Martinu T, Todd JL, Gelman AE, Guerra S, Palmer SM. Club cell secretory protein in lung disease: emerging concepts and potential therapeutics. Annu Rev Med. 2023;74:427–41. 10.1146/annurev-med-042921-123443. 36450281 10.1146/annurev-med-042921-123443 PMC10472444

[CR44] Metcalfe C, Kljavin NM, Ybarra R, de Sauvage FJ. Lgr5+ stem cells are indispensable for radiation-induced intestinal regeneration. Cell Stem Cell. 2014;14(2):149–59. 10.1016/j.stem.2013.11.008. 24332836 10.1016/j.stem.2013.11.008

[CR45] Montoro DT, Haber AL, Biton M, Vinarsky V, Lin B, Birket SE, et al. A revised airway epithelial hierarchy includes CFTR-expressing ionocytes. Nature. 2018;560(7718):319–24. 10.1038/s41586-018-0393-7. 30069044 10.1038/s41586-018-0393-7 PMC6295155

[CR46] Polański K, Young MD, Miao Z, Meyer KB, Teichmann SA, Park J-E. BBKNN: fast batch alignment of single cell transcriptomes. Bioinforma Oxf Engl. 2020;36(3):964–5. 10.1093/bioinformatics/btz625. 10.1093/bioinformatics/btz625 PMC9883685 31400197

[CR47] Potter SS. Single-cell RNA sequencing for the study of development, physiology and disease. Nat Rev Nephrol. 2018;14(8):479–92. 10.1038/s41581-018-0021-7. 29789704 10.1038/s41581-018-0021-7 PMC6070143

[CR48] Rimland CA, Tilson SG, Morell CM, Tomaz RA, Lu W-Y, Adams SE, et al. Regional differences in human biliary tissues and corresponding in vitro-derived organoids. Hepatology. 2021;73(1):247–67. 10.1002/hep.31252. 32222998 10.1002/hep.31252 PMC8641381

[CR49] Rock JR, Onaitis MW, Rawlins EL, Lu Y, Clark CP, Xue Y, et al. Basal cells as stem cells of the mouse trachea and human airway epithelium. Proc Natl Acad Sci U S A. 2009;106(31):12771–5. 10.1073/pnas.0906850106. 19625615 10.1073/pnas.0906850106 PMC2714281

[CR50] Ross AA, Cooper BW, Lazarus HM, Mackay W, Moss TJ, Ciobanu N, et al. Detection and viability of tumor cells in peripheral blood stem cell collections from breast cancer patients using immunocytochemical and clonogenic assay techniques. Blood. 1993;82(9):2605–10. 8219214

[CR51] Ruan X, Kang B, Qi C, Lin W, Wang J, Zhang X. Progenitor cell diversity in the developing mouse neocortex. Proc Natl Acad Sci U S A. 2021;118(10):e2018866118. 10.1073/pnas.2018866118. 33649223 10.1073/pnas.2018866118 PMC7958455

[CR52] Saillour Y, Broix L, Bruel-Jungerman E, Lebrun N, Muraca G, Rucci J, et al. Beta tubulin isoforms are not interchangeable for rescuing impaired radial migration due to Tubb3 knockdown. Hum Mol Genet. 2014;23(6):1516–26. 10.1093/hmg/ddt538. 24179174 10.1093/hmg/ddt538

[CR53] Sang Q, Kong F. Applications for single-cell and spatial transcriptomics in plant research. New Crops. 2024;1:100025. 10.1016/j.ncrops.2024.100025.

[CR54] Sekine Y, Algarate PT, Cafferty WBJ, Strittmatter SM. Plexina2 and CRMP2 signaling complex is activated by Nogo-A-liganded Ngr1 to restrict corticospinal axon sprouting after trauma. J Neurosci off J Soc Neurosci. 2019;39(17):3204–16. 10.1523/JNEUROSCI.2996-18.2019. 10.1523/JNEUROSCI.2996-18.2019 PMC6788813 30804090

[CR55] Shaffer SM, Dunagin MC, Torborg SR, Torre EA, Emert B, Krepler C, et al. Rare cell variability and drug-induced reprogramming as a mode of cancer drug resistance. Nature. 2017;546(7658):431–5. 10.1038/nature22794. 28607484 10.1038/nature22794 PMC5542814

[CR56] Si J, Su X, Jin Z, Duan S. Uncovering essential lncRNAs through transcriptome-scale CRISPR-Cas13 screening. Adv Biotechnol. 2025;3(3):27. 10.1007/s44307-025-00082-8. 10.1007/s44307-025-00082-8 PMC12436244 40952596

[CR57] Srivatsan SR, Regier MC, Barkan E, Franks JM, Packer JS, Grosjean P, et al. Embryo-scale, single-cell spatial transcriptomics. Science. 2021;373(6550):111–7. 10.1126/science.abb9536. 34210887 10.1126/science.abb9536 PMC9118175

[CR58] Suzuki Y, Lutshumba J, Chen KC, Abdelaziz MH, Sa Q, Ochiai E. IFN-γ production by brain-resident cells activates cerebral mRNA expression of a wide spectrum of molecules critical for both innate and T cell-mediated protective immunity to control reactivation of chronic infection with Toxoplasma gondii. Front Cell Infect Microbiol. 2023;13:1110508. 10.3389/fcimb.2023.1110508. 36875520 10.3389/fcimb.2023.1110508 PMC9975934

[CR59] Tamura K, Yu J, Hata T, Suenaga M, Shindo K, Abe T, et al. Mutations in the pancreatic secretory enzymes CPA1 and CPB1 are associated with pancreatic cancer. Proc Natl Acad Sci U S A. 2018;115(18):4767–72. 10.1073/pnas.1720588115. 29669919 10.1073/pnas.1720588115 PMC5939087

[CR60] Tang S, Huang X, Wang X, Zhou X, Huang H, Qin L, et al. Vital and distinct roles of H2A.Z isoforms in hepatocellular carcinoma. OncoTargets Ther. 2020;13:4319–37. 10.2147/OTT.S243823. 10.2147/OTT.S243823 PMC7244249 32547065

[CR61] Tao S, Tang D, Morita Y, Sperka T, Omrani O, Lechel A, et al. Wnt activity and basal niche position sensitize intestinal stem and progenitor cells to DNA damage. EMBO J. 2015;34(5):624–40. 10.15252/embj.201490700. 25609789 10.15252/embj.201490700 PMC4365032

[CR62] Valente M, Collinet N, Vu Manh T-P, Popoff D, Rahmani K, Naciri K, et al. Novel mouse models based on intersectional genetics to identify and characterize plasmacytoid dendritic cells. Nat Immunol. 2023;24(4):714–28. 10.1038/s41590-023-01454-9. 36928414 10.1038/s41590-023-01454-9 PMC10063451

[CR63] Wang X, Duan M, Li J, Ma A, Xin G, Xu D, et al. MarsGT: Multi-omics analysis for rare population inference using single-cell graph transformer. Nat Commun. 2024;15(1):338. 10.1038/s41467-023-44570-8. 38184630 10.1038/s41467-023-44570-8 PMC10771517

[CR64] Wegmann R, Neri M, Schuierer S, Bilican B, Hartkopf H, Nigsch F, et al. CellSIUS provides sensitive and specific detection of rare cell populations from complex single-cell RNA-seq data. Genome Biol. 2019;20(1):142. 10.1186/s13059-019-1739-7. 31315641 10.1186/s13059-019-1739-7 PMC6637521

[CR65] Wijnands C, Noori S, Donk NWCJ, VanDuijn MM, Jacobs JFM. Advances in minimal residual disease monitoring in multiple myeloma. Crit Rev Clin Lab Sci. 2023;60(7):518–34. 10.1080/10408363.2023.2209652. 37232394 10.1080/10408363.2023.2209652

[CR66] Xu Y, Wang S, Feng Q, Xia J, Li Y, Li H-D, et al. scCAD: Cluster decomposition-based anomaly detection for rare cell identification in single-cell expression data. Nat Commun. 2024;15(1):7561. 10.1038/s41467-024-51891-9. 39215003 10.1038/s41467-024-51891-9 PMC11364754

[CR67] Yong CH, Hoon S, De Mel S, Xu S, Scolnick JA, Huo X, et al. Mapbatch: conservative batch normalization for single-cell RNA-sequencing data enables discovery of rare cell populations in a multiple myeloma cohort. Blood. 2021;138(Supplement 1):2954–2954. 10.1182/blood-2021-150089.

[CR68] Yu Z, Su Y, Lu Y, Yang Y, Wang F, Zhang S, et al. Topological identification and interpretation for single-cell gene regulation elucidation across multiple platforms using scMGCA. Nat Commun. 2023;14(1):400. 10.1038/s41467-023-36134-7. 36697410 10.1038/s41467-023-36134-7 PMC9877026

[CR69] Zappia L, Phipson B, Oshlack A. Splatter: simulation of single-cell RNA sequencing data. Genome Biol. 2017;18(1):174. 10.1186/s13059-017-1305-0. 28899397 10.1186/s13059-017-1305-0 PMC5596896

[CR70] Zhang G, He P, Tan H, Budhu A, Gaedcke J, Ghadimi BM, et al. Integration of metabolomics and transcriptomics revealed a fatty acid network exerting growth inhibitory effects in human pancreatic cancer. Clin Cancer Res off J Am Assoc Cancer Res. 2013;19(18):4983–93. 10.1158/1078-0432.CCR-13-0209. 10.1158/1078-0432.CCR-13-0209 PMC3778077 23918603

[CR71] Zhang Y, Parmigiani G, Johnson WE. ComBat-seq: batch effect adjustment for RNA-seq count data. NAR Genom Bioinform. 2020;2(3):lqaa078. 10.1093/nargab/lqaa078. 33015620 10.1093/nargab/lqaa078 PMC7518324

[CR72] Zhang Y, Narayanan SP, Mannan R, Raskind G, Wang X, Vats P, et al. Single-cell analyses of renal cell cancers reveal insights into tumor microenvironment, cell of origin, and therapy response. Proc Natl Acad Sci U S A. 2021;118(24):e2103240118. 10.1073/pnas.2103240118. 34099557 10.1073/pnas.2103240118 PMC8214680

[CR73] Zhou S, Li Y, Wu W, Li L. ScMMT: a multi-use deep learning approach for cell annotation, protein prediction and embedding in single-cell RNA-seq data. Brief Bioinform. 2024;25(2):bbad523. 10.1093/bib/bbad523. 38300515 10.1093/bib/bbad523 PMC10833085

