# Supplementary material for: Specificity-driven cell-gene graph learning identifies rare cell states in single-cell and spatial transcriptomic data
Source: Adv Biotechnol (Singap). 2026 Jul 16;4(3):26. doi: 10.1007/s44307-026-00121-y (PMC13376285; doi:10.1007/s44307-026-00121-y)
Supplement: Supplementary file 1 — Supplementary Material 1. [file 44307_2026_121_MOESM1_ESM.docx]

**Supplementary Information** for

# Specificity-driven cell-gene graph learning identifies rare cell states in single-cell and spatial transcriptomic data

Jinjin Huang^1,2^, Xuanzhe Xia^2^, Feng Luo^1^, Lianghu Qu^2^, Xiao Feng^1,*^, Lingling Zheng^1,2,*^

^1^ School of Agriculture and Biotechnology, Sun Yat-sen University Shenzhen Campus, Shenzhen 518107, China

^2^ MOE Key Laboratory of Gene Function and Regulation, State Key Laboratory for Biocontrol, Guangdong Provincial Key Laboratory of Plant Stress, Innovation Center for Evolutionary Synthetic Biology, School of Life Sciences, Sun Yat-sen University, Guangzhou 510275, China

## Supplementary Figures


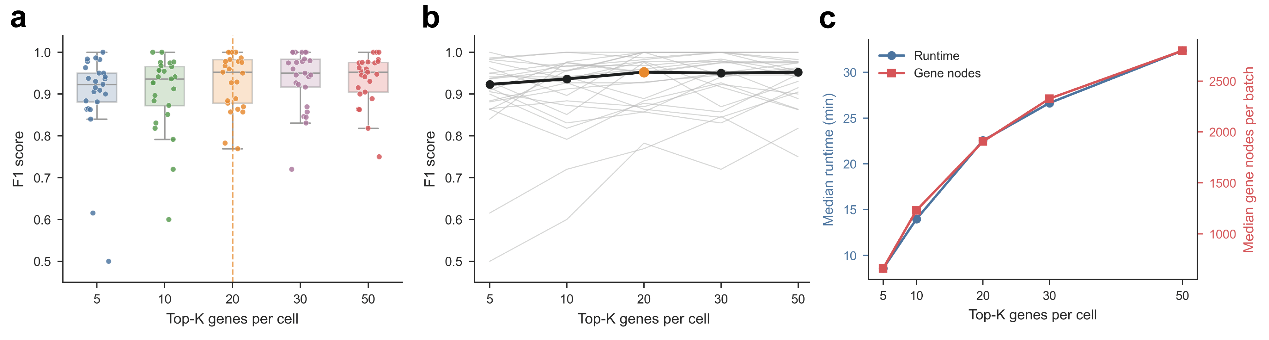


**Supplementary Fig. 1 | Sensitivity of scFormer to the graph-sparsity parameter K.** **a**, Distribution of F1 scores across 25 simulated datasets when *K* was set to 5, 10, 20, 30 or 50. The dashed line marks the default value *K* = 20 used in the main analyses. **b**, Paired dataset-level F1 trajectories across *K* values, with the black line indicating the median F1 score. **c**, Median runtime and median number of gene nodes per batch as *K* increases. Performance increased from smaller *K* values to *K* = 20 and then approached a plateau, whereas runtime and graph size continued to increase with larger *K* values.


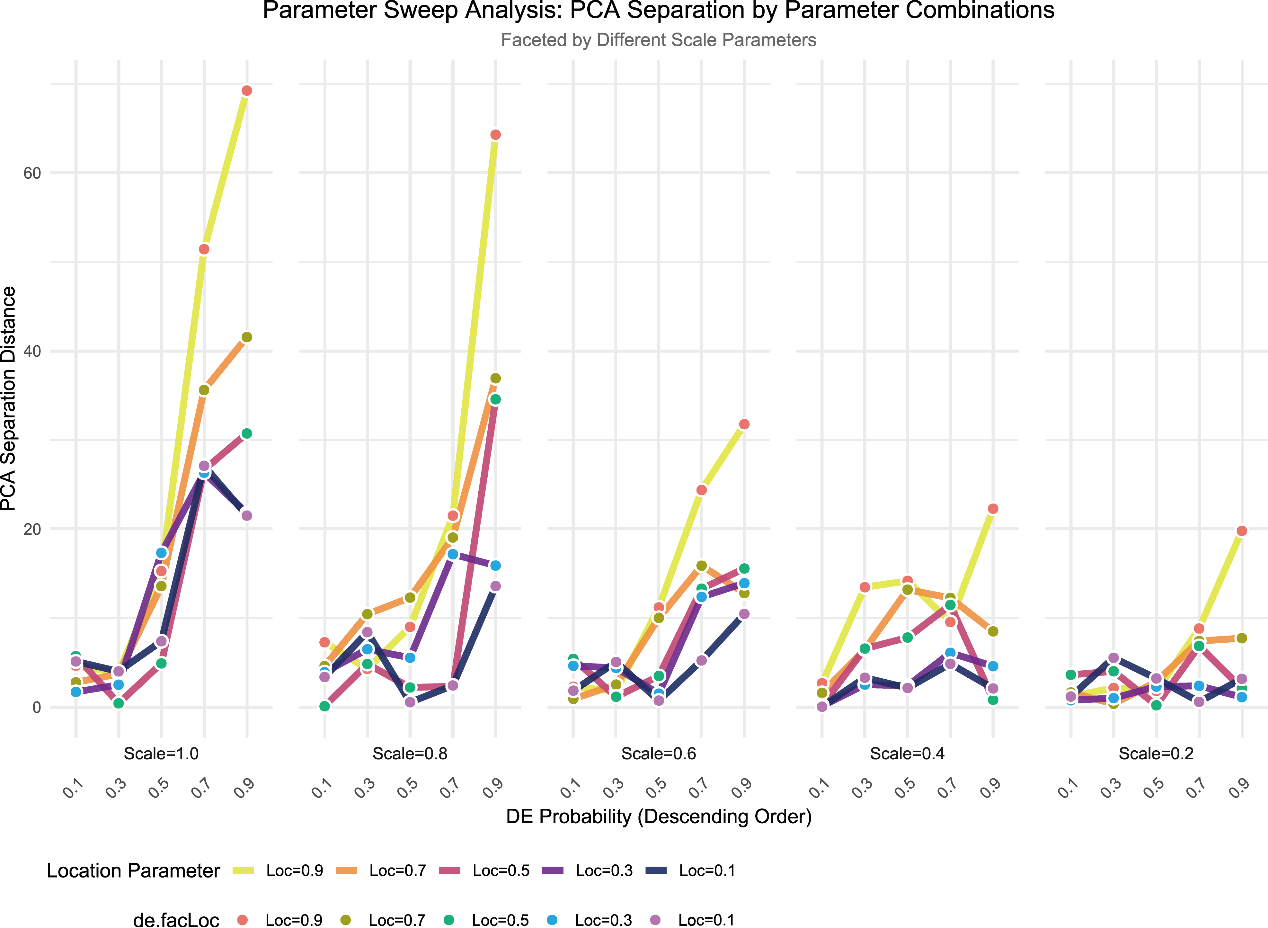


**Supplementary Fig. 2** | **Impact of simulation parameters on cluster separability in simulated datasets.** To assess the ability to distinguish between cell populations under challenging conditions, we generated 125 simulated datasets using the splatter framework. Each dataset was configured with two groups at a highly imbalanced ratio of 99:1. We defined a "separation index" to quantify the distinction between the groups, calculated as the Euclidean distance between the cluster centroids in the principal component (PC) space. This figure illustrates how the separation index varies as a function of three key splatter parameters: (1) the proportion of differentially expressed (DE) genes (de.prob), (2) the location parameter of DE fold changes (de.facLoc), and (3) the scale parameter of DE fold change dispersion (de.facScale).


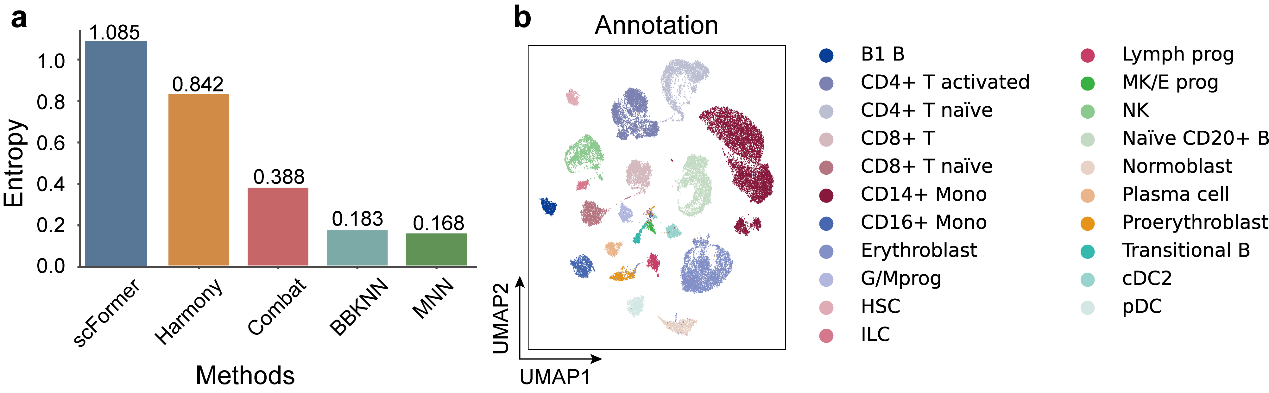


**Supplementary Fig. 3 | Identification of rare cell populations (< 1% abundance) in the GSE194122** **PBMC dataset.** **a,** Quantitative evaluation of batch correction performance. scFormer achieves the highest batch entropy of 1.085, surpassing Harmony (0.842) and other benchmarked methods, which signifies more effective batch integration. **b,** UMAP visualizations of multi-sample GSE194122 PBMC dataset, colored by original cell type annotations.


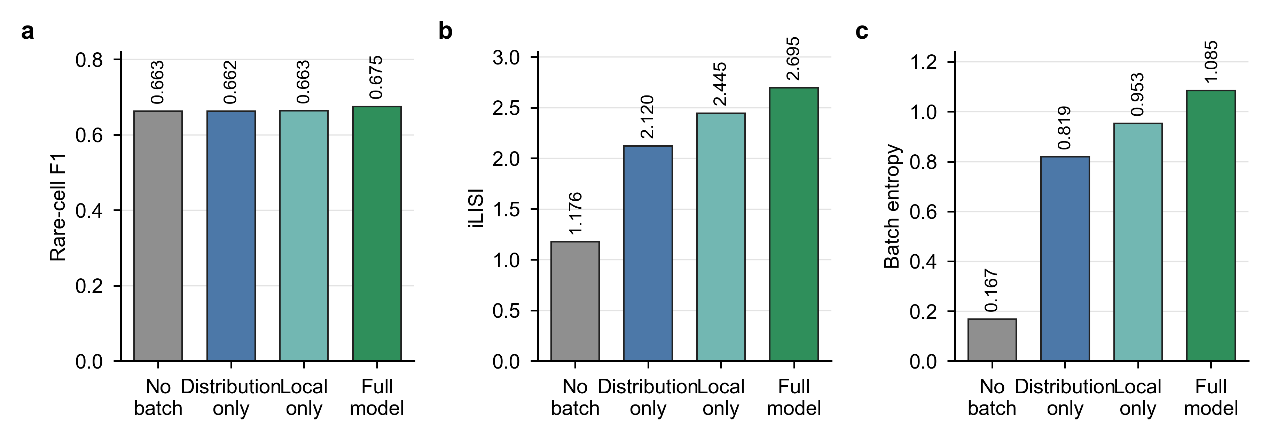


**Supplementary Fig. 4 | Component ablation of the scFormer batch-correction loss in the PBMC analysis.** **a**, Rare-cell F1 scores under four settings: no batch correction, distribution-component-only, local-structure-component-only, and the full scFormer model. The distribution component denotes the batch-level embedding moment-alignment term, whereas the local-structure component denotes the nearest-neighbor structure-preservation term described in Methods. Rare-cell F1 was computed by comparing predicted rare cells with reference rare labels derived from the original study annotations. **b**, iLISI values across the same four settings, quantifying local batch mixing. **c**, Batch entropy values across the same four settings. Values above bars indicate the corresponding metric values. The component ablation shows that batch-correction components improve batch mixing while maintaining rare-cell recovery, with the full model achieving the strongest overall integration performance.


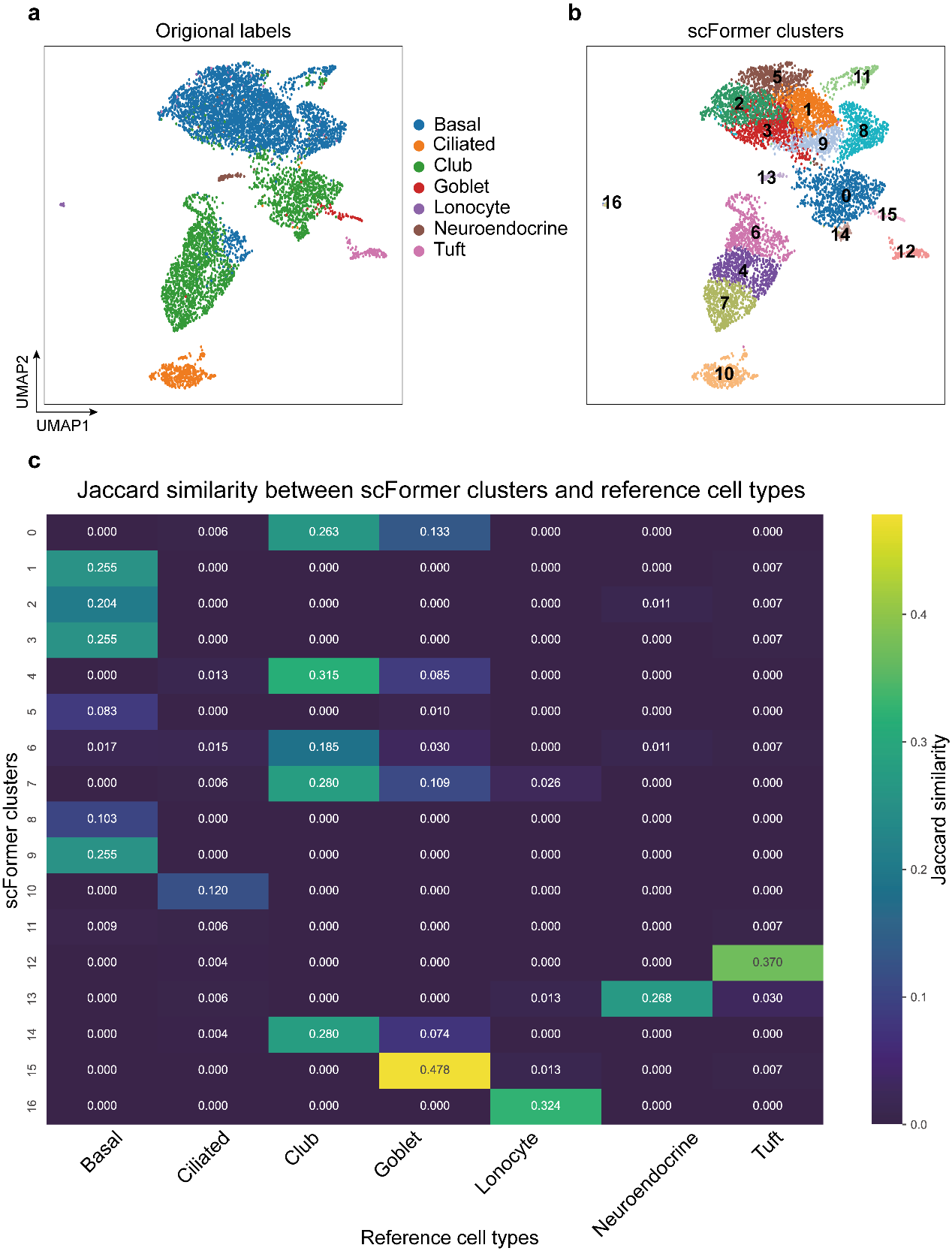


**Supplementary Fig. 5 | Annotation of scFormer-defined clusters in mouse airway epithelial cells via Jaccard similarity.** **a**, **b**, UMAP visualizations of 7,193 mouse airway epithelial cells, colored by (**a**) original cell type annotations and (**b**) the 17 clusters identified by scFormer. **c,** This heatmap displays the Jaccard similarity coefficients computed between each scFormer-defined cluster and the seven reference annotated cell types from the mouse airway epithelium dataset. The similarity was calculated based on the overlap between the top 50 marker genes identified for each scFormer cluster and the established marker genes for the annotated cell types derived from the original study. The resulting scores facilitate the assignment of biological cell type identities to the computationally derived clusters, with higher values (indicated by color intensity) suggesting a stronger correspondence.


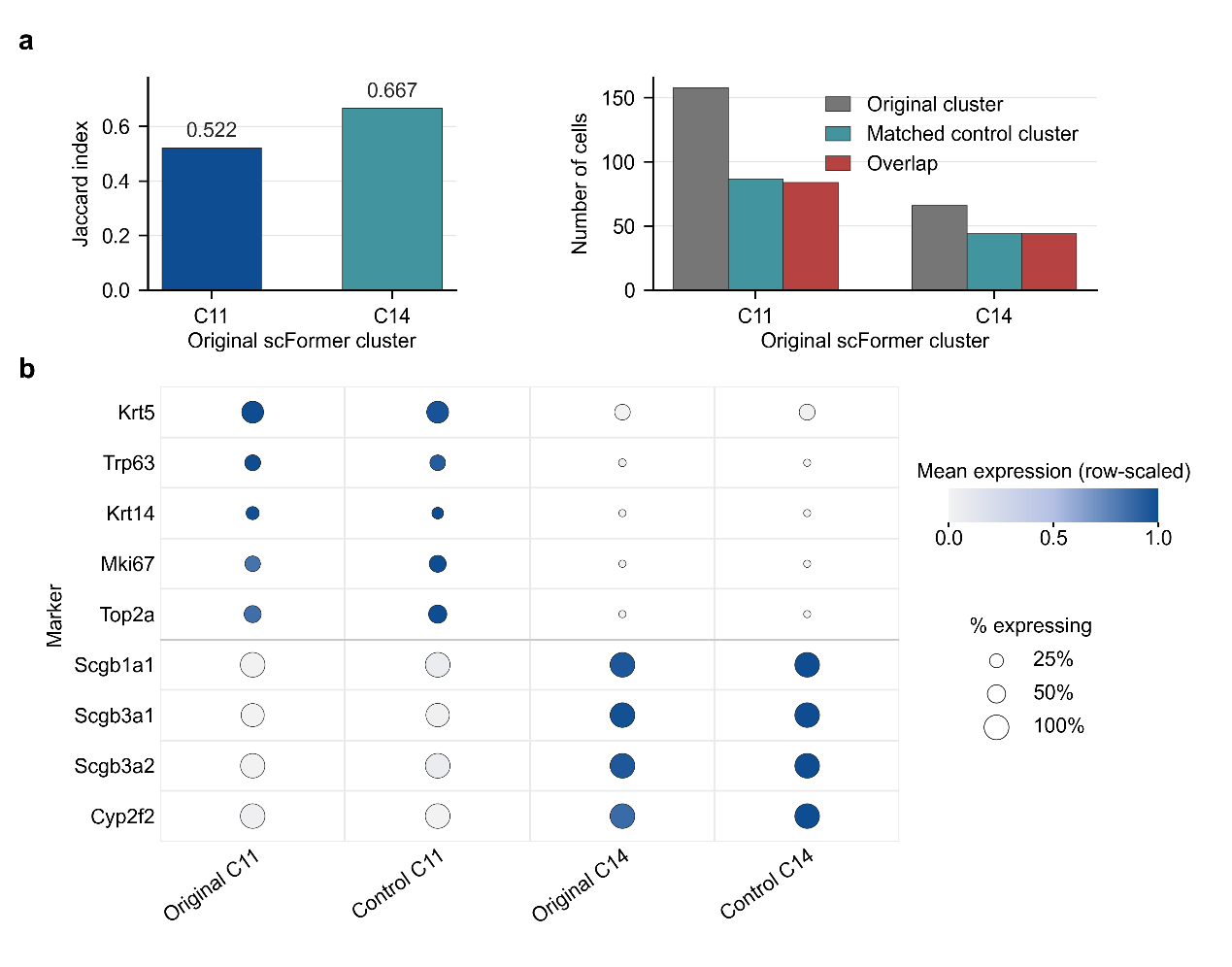


**Supplementary Fig. 6 | Cell-cycle gene-removal control for airway rare clusters. a,** Quantitative overlap between the original scFormer rare clusters and their best-matched clusters after rerunning scFormer on the Airway dataset with 94 canonical murine S/G2M genes removed. Left, Jaccard index between original clusters 11 and 14 and their matched control clusters. Right, original cluster size, matched control cluster size, and overlapping cell counts. **b,** Dot plot showing marker retention in the original clusters and the corresponding matched clusters after S/G2M gene removal. Dot size indicates the percentage of cells expressing each marker, and color indicates row-scaled mean expression. Basal-associated markers (*Krt5*, *Trp63*, *Krt14*), cell-cycle-associated markers (*Mki67*, *Top2a*), and club-cell-associated markers (*Scgb1a1*, *Scgb3a1*, *Scgb3a2*, *Cyp2f2*) are shown.


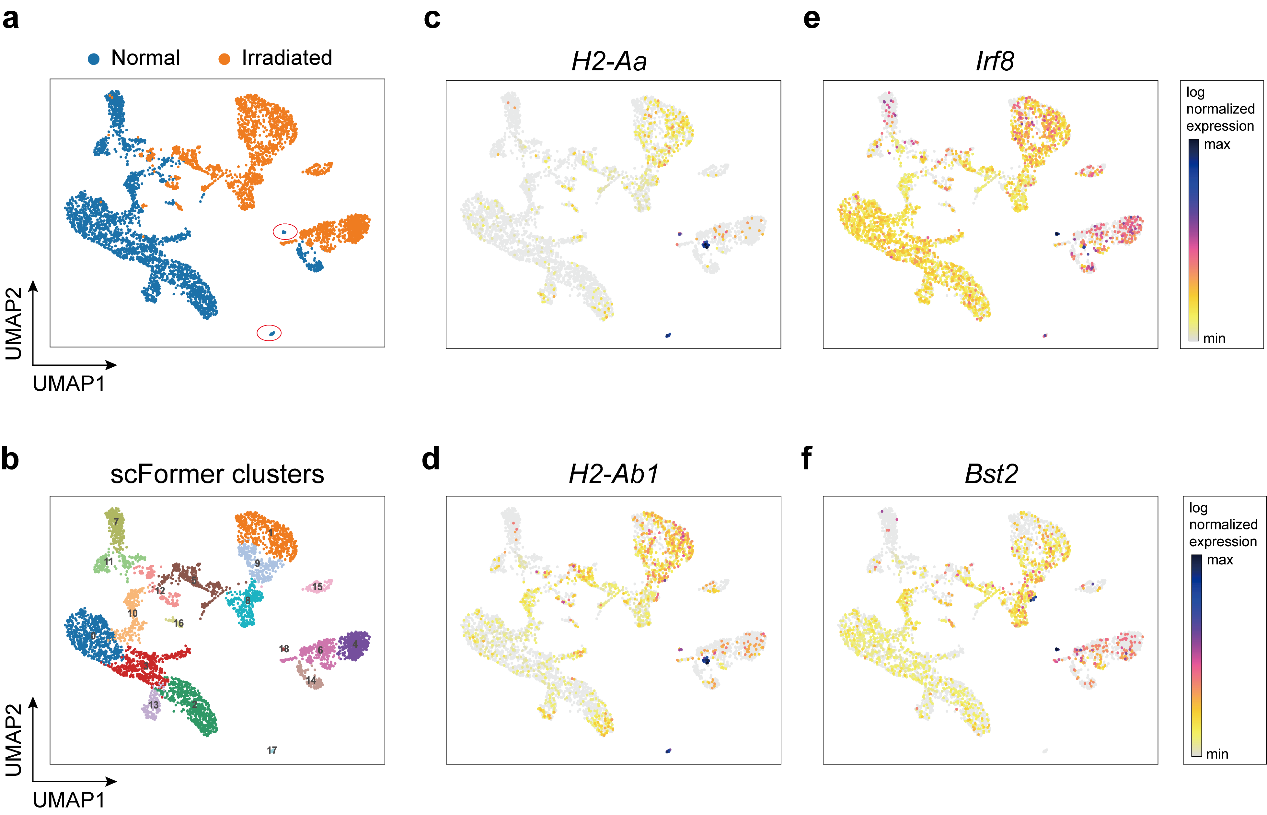


**Supplementary Fig. 7 | Analysis of single-cell RNA sequencing data from the mouse intestinal epithelium, comparing normal and irradiated samples.** **a**, UMAP visualization of all cells, colored by experimental condition (Normal or Irradiated). **b**, The same UMAP visualization, colored by the distinct clusters identified by scFormer. **c-f**, UMAP feature plots displaying the normalized expression of key genes associated with antigen presentation and interferon response: *H2-Aa* (**c**), *H2-Ab1* (**d**), *Irf8* (**e**), and *Bst2* (**f**). The color scale represents the level of gene expression, from low (gray) to high (red).


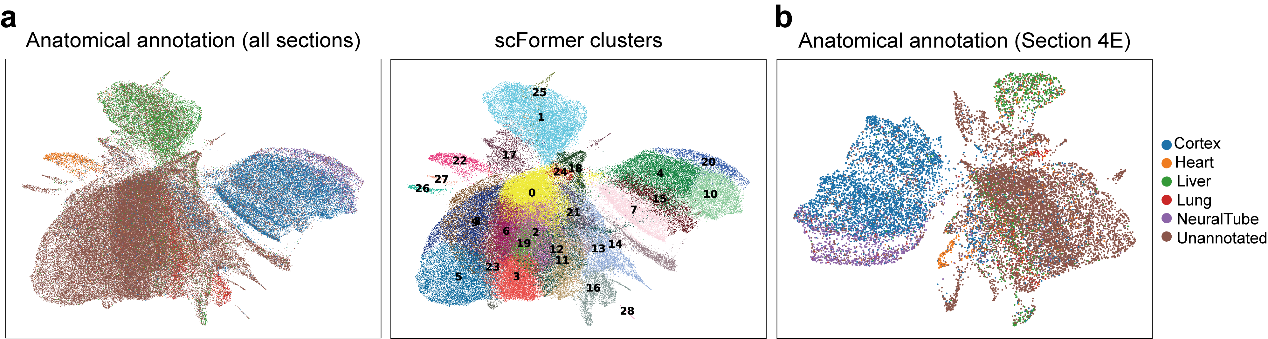


**Supplementary Fig. 8 | UMAP-based clustering of the sci-Space E14 mouse embryo dataset.** **a**, UMAP visualizations of all spots aggregated from all available tissue sections. The left panel is colored by the original annotations, and the right panel is colored by scFormer clusters. **b**, UMAP visualization of spots from a single section (slide_4E), colored by the original annotations.


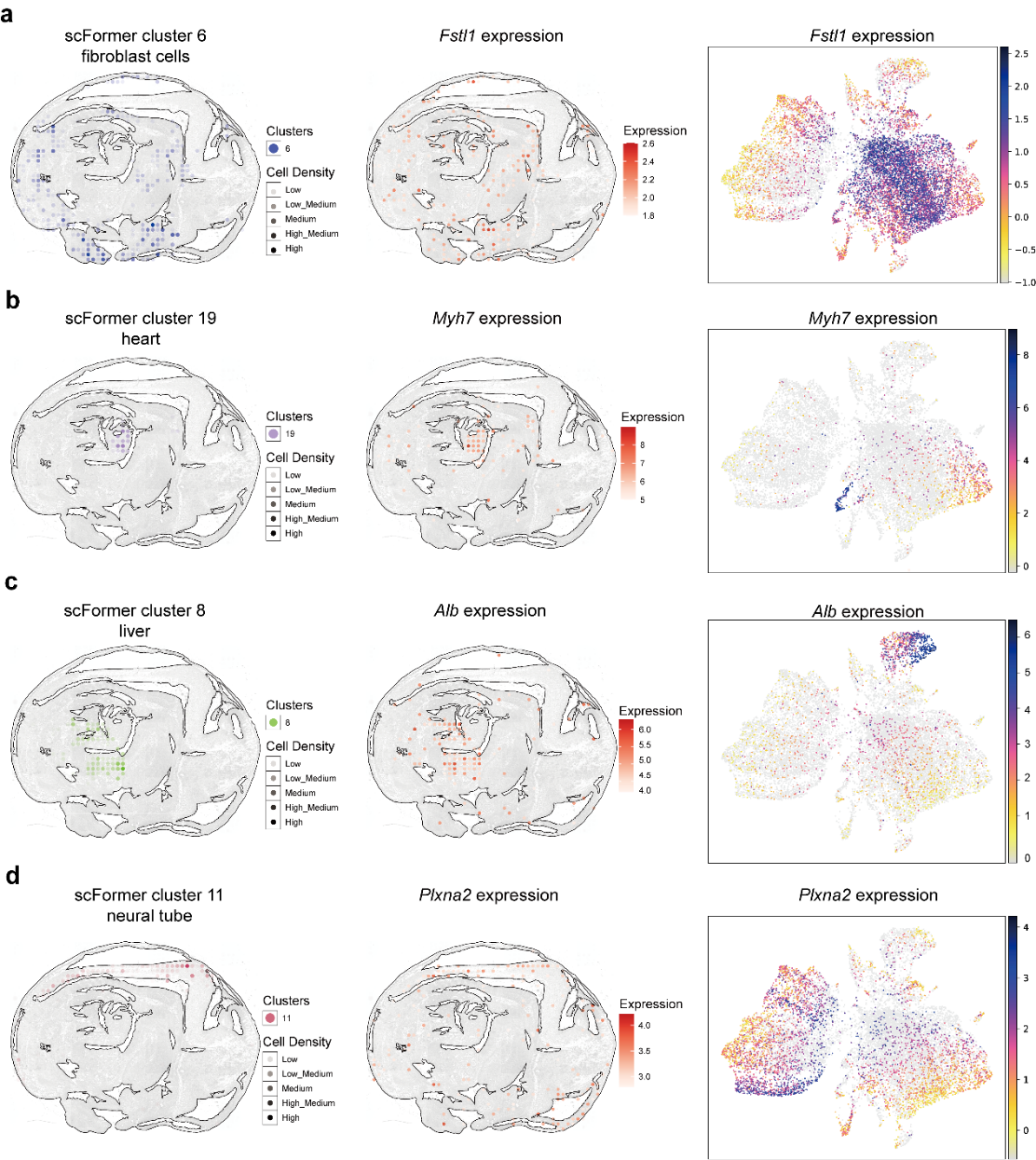


**Supplementary Fig. 9 | Spatial and UMAP visualization of key cell populations in mouse embryo slice slide_4E.** All panels visualize data from a single representative sagittal section (slide_4E, n = 16,222 spots) of the sci-Space E14 mouse embryo dataset. This figure demonstrates the correspondence between scFormer-defined clusters, their spatial locations, and the expression of canonical marker genes. For each cell type shown, the panels display (from left to right): the spatial distribution of the identified cluster, the spatial expression of its marker gene, and the marker's expression on the UMAP projection. **a**, Identification of a widespread fibroblast population, marked by *Fstl1*. **b**, Identification of the organ-specific cardiac muscle population, marked by *Myh7*. **c**, Identification of the organ-specific hepatic cell population, marked by *Alb*. **d**, Identification of the organ-specific spinal cord population, marked by *Plxna2*.


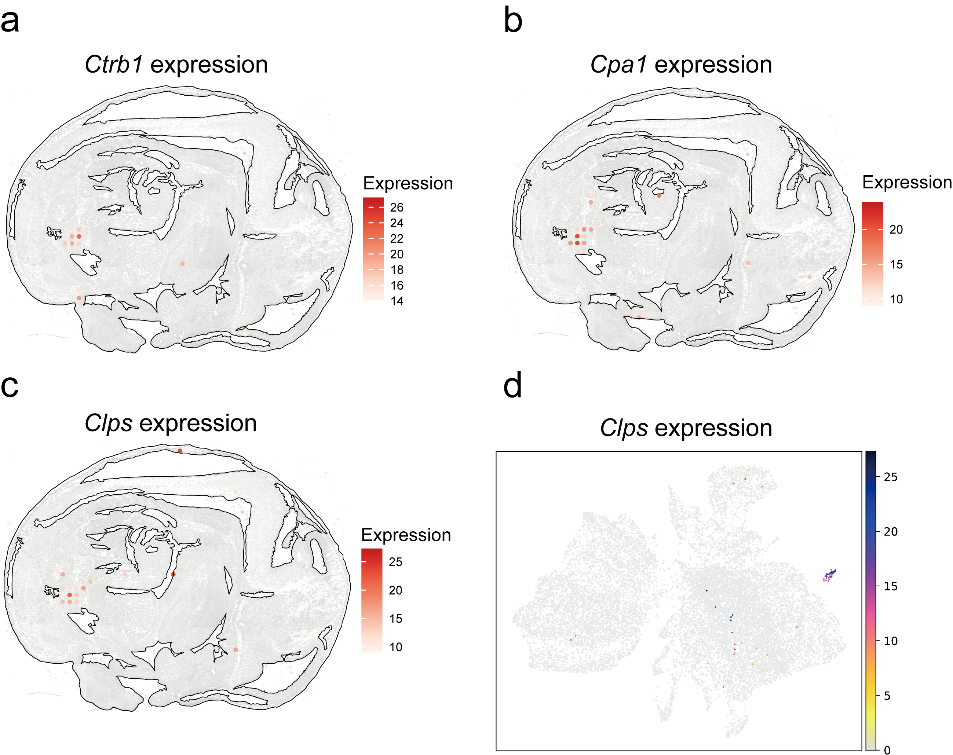


**Supplementary Fig. 10 | Marker visualization for pancreatic-associated rare cells in slide_4E.** This figure provides additional marker-level evidence for the pancreatic marker-enriched rare cell state identified in the E14 mouse embryo section. a-c, Spatial feature plots showing expression of Ctrb1 (a), Cpa1 (b), and Clps (c), three pancreatic marker genes. d, UMAP feature plot for Clps, showing that Clps expression is concentrated in the same expression-space region as the cluster highlighted in Fig. 7c-d. These marker visualizations complement the quantitative enrichment analyses shown in Supplementary Fig. 11.


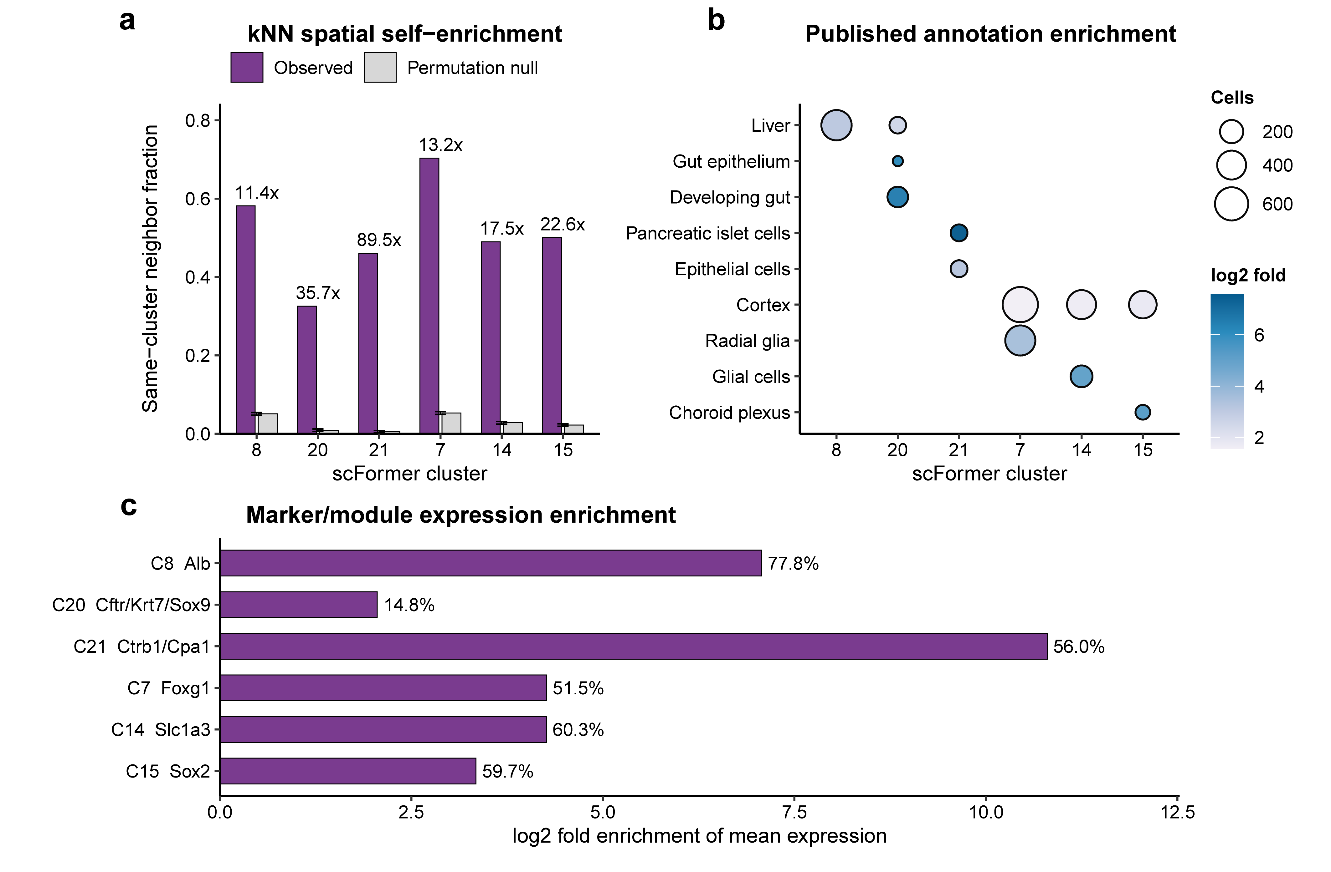


**Supplementary Fig. 11 | Quantitative evaluation of spatial organization, published annotation concordance, and marker enrichment for selected scFormer clusters in slide_4E.** **a**, k-nearest-neighbor spatial self-enrichment analysis. For each selected cluster, bars show the fraction of same-cluster cells among the 10 nearest spatial neighbors; gray bars show the mean size-matched permutation background from 10,000 permutations. Numbers above bars indicate fold enrichment over the permutation background. **b**, Hypergeometric enrichment of selected scFormer clusters against published sci-Space annotation categories from anatomical_annotation, manual_annotation_2, and final_cluster_label. Dot size indicates the number of cells overlapping each annotation category, and color indicates log2 fold enrichment. **c**, Marker or marker-module expression enrichment for selected cell states. Bars show log2 fold enrichment of mean marker or marker-module expression in the indicated cluster relative to all other cells. Percentages indicate the fraction of cells in that cluster expressing the marker or co-expressing all genes in the marker module.


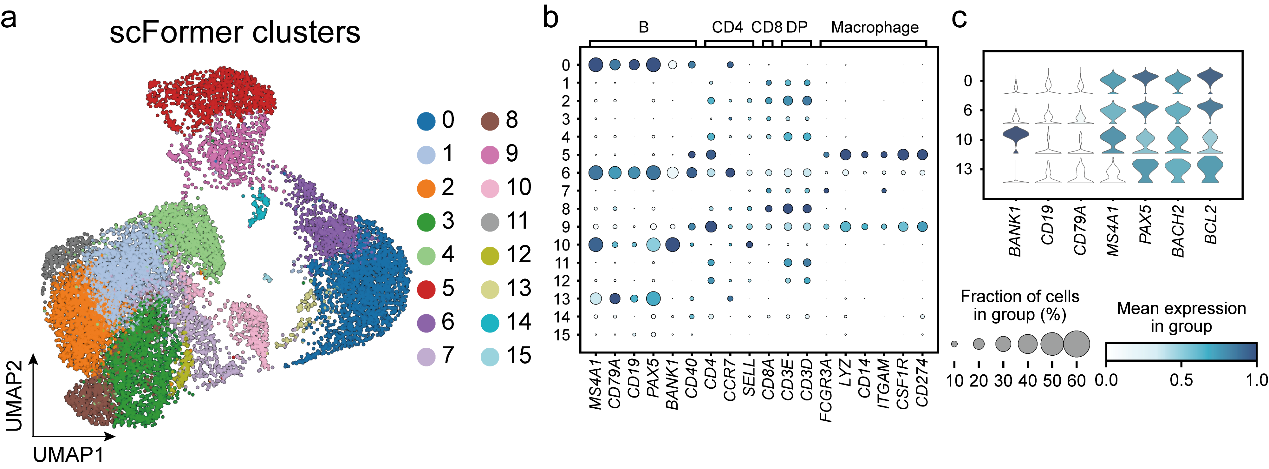


**Supplementary Fig. 12 | scFormer identifies a rare B cell state from RNA data alone.** **a,** UMAP visualization of scFormer clusters inferred from the matched lymphoma scRNA seq dataset, with colors indicating distinct cell clusters. **b,** Dot plot showing the expression of canonical marker genes across annotated immune cell groups (B, CD4 T, CD8 T, DP, and macrophage). Dot size denotes the fraction of cells expressing each gene within a group, and color intensity represents mean expression. **c,** Violin plots illustrating the expression distributions of representative B cell markers across selected clusters, highlighting a rare B cell population (cluster 13). In relation to recent multimodal rare state discovery frameworks such as MarsGT, which reported that a rare B lymphoma precursor like state (BLS1) could only be resolved through joint RNA ATAC modeling, scFormer recovers an analogous rare B cell state using the RNA modality alone (cluster 13).

## Supplementary Tables

**Supplementary Table 1** Benchmarking scope, parameter settings, rare-cell calling rules, and tuning strategy for scFormer and comparator methods.

| Method | Benchmark role | Parameterization and rare-cell calling |
| --- | --- | --- |
| scFormer | Proposed method; rare-cell identification with optional batch correction | Expression matrix represented as a cell-gene heterogeneous graph. Initial pseudo-labels were generated by Leiden clustering on normalized and log-transformed expression profiles. Main settings: dataset-size-dependent Leiden initialization (<=500 cells: resolution 0.2, 5 neighbors; 500-5,000 cells: resolution 0.5, 10 neighbors; >5,000 cells: resolution 0.8, 15 neighbors), hidden dimension 104, 8 attention heads, 3 layers, label smoothing 0.1, learning rate 0.0005, weight decay 0.1, and 100 epochs. Predicted clusters with abundance <=5% were counted as rare. Parameters were fixed before evaluation and were not optimized using reference rare-cell labels. |
| CellSIUS | Rare-cell and subcluster detection comparator | Seurat-normalized expression matrix with clusters from the standard Seurat workflow. CellSIUS settings: min_n_cells=10, min_fc=1, corr_cutoff=NULL, iter=0, max_perc_cells=5, and fc_between_cutoff=2. Cells assigned to nonzero CellSIUS subclusters were counted as rare. Parameters followed the documented CellSIUS workflow and were fixed before evaluation. |
| FiRE | Rarity-score-based comparator | Normalized cell-by-gene expression matrix. FiRE settings: L=100, M=50, H=1017881, seed=5489, verbose=0. FiRE scores were thresholded using Q3 + 1.5 x IQR, and cells above this threshold were counted as rare. The model settings and thresholding rule were fixed before evaluation. |
| GapClust | K-nearest-neighbor gap-based comparator | Seurat-normalized expression matrix followed by variable-feature selection, log2(x+1) transformation, and PCA. GapClust settings: k=200 nearest neighbors, up to 50 principal components, and skewness >2 for candidate gap selection. Cells returned by the GapClust rare-cell index output were counted as rare. KNN and skewness settings were fixed before evaluation. |
| GiniClust3 | Gini/Fano/consensus-based comparator | Cells with fewer than 3 detected genes and genes detected in fewer than 200 cells were filtered; counts were normalized to 10,000 per cell. GiniClust3 used the standard Gini, Fano, and consensus workflow; the Gini clustering step used neighbors=3. Final consensus clusters containing <5% of cells were counted as rare. Package workflow settings were fixed before evaluation. |
| RaceID3 | Clustering and outlier-based comparator | Count matrix used to construct SCseq objects after low-quality cell filtering. RaceID3 settings: filterdata(mintotal=1000, minexpr=5, minnumber=5), Pearson distance, clustexp(cln=10, sat=TRUE, bootnr=50, FUNcluster="kmeans"), and findoutliers. Clusters with size below 5% of the total cell number were counted as rare. Settings were fixed before evaluation. |
| scCAD | Cluster decomposition-based anomaly-detection comparator | Expression matrix with internal gene filtering, per-cell normalization, log transformation, and highly variable gene selection. scCAD settings: seed=2023, merge_h=50, overlap_h=0.7, rare_h=0.05, and internal fast-clustering k=15. Cells returned by scCAD rare-cell clusters were counted as rare. Published/default-style settings were fixed before evaluation. |
| SCMER | Feature-learning comparator followed by clustering | Cells with fewer than 3 detected genes and genes detected in fewer than 200 cells were filtered; counts were normalized to 10,000 per cell and log-transformed. SCMER settings: target_n_features=500, perplexity=30, smallest_log10_fold_change=-3, max_iter=10, use_gpu=True, followed by KMeans clustering. KMeans clusters with abundance <5% were counted as rare. Settings were fixed before evaluation. |
| Harmony | Batch-correction comparator | PBMC expression profiles normalized, log-transformed, scaled, projected by PCA, and corrected by Harmony using batch labels. Harmony was applied to PCA embeddings, and downstream nearest-neighbor graph construction used Harmony-corrected PCA embeddings with 10 neighbors. Harmony alone was evaluated for batch correction; rare-cell calling was assessed through the Harmony-Leiden workflow. |
| BBKNN | Graph-based batch-alignment comparator | Applied to the PBMC AnnData object after common preprocessing and PCA. BBKNN was run with batch_key="batch" using package defaults, followed by UMAP and Leiden clustering for visualization and metric calculation. BBKNN was evaluated for batch correction and was not used as a standalone rare-cell detector in the main rare-cell benchmark. |
| MNN Correct | Mutual-nearest-neighbor batch-correction comparator | PBMC expression profiles corrected using batch labels, followed by PCA, nearest-neighbor graph construction, UMAP, and Leiden clustering. mnn_correct was run with batch_key="batch"; downstream graph construction used n_neighbors=10 on PCA embeddings. MNN Correct was evaluated for batch correction, not as a standalone rare-cell detector. |
| ComBat | Batch-correction comparator | PBMC expression profiles corrected using batch labels, followed by PCA, nearest-neighbor graph construction, UMAP, and Leiden clustering. ComBat was run with key="batch"; downstream graph construction used n_neighbors=10 on PCA embeddings. ComBat was evaluated for batch correction, not as a standalone rare-cell detector. |
| Harmony-Leiden | Two-stage integration-plus-clustering baseline for PBMC rare-cell recovery | PBMC expression profiles normalized, log-transformed, scaled, projected by PCA, corrected by Harmony using batch labels, and clustered using a nearest-neighbor graph built from Harmony-corrected embeddings. The main comparison used a predefined Harmony-Leiden setting. To assess resolution sensitivity, Leiden resolutions of 0.2, 0.4, 0.6, 0.8, 1.0, 1.2, 1.5, 2.0, 2.5, and 3.0 were additionally evaluated. Clusters with abundance <5% were counted as predicted rare clusters; full sweep results are reported separately in the Harmony-Leiden resolution-sensitivity table. |

**Supplementary Table 2** Harmony-Leiden resolution-sensitivity analysis in the PBMC multi-sample dataset. Reference rare cells were defined from the original annotations using the 5% prevalence threshold (n = 7,545).

| Leiden resolution | Number of clusters | Precision | Recall | F1 score | Predicted rare cells |
| --- | --- | --- | --- | --- | --- |
| 0.2 | 12 | 0.909 | 0.525 | 0.666 | 4,360 |
| 0.4 | 16 | 0.877 | 0.672 | 0.761 | 5,784 |
| 0.6 | 20 | 0.864 | 0.885 | 0.874 | 7,724 |
| 0.8 | 23 | 0.840 | 0.889 | 0.864 | 7,984 |
| 1.0 | 25 | 0.817 | 0.909 | 0.861 | 8,393 |
| 1.2 | 28 | 0.522 | 0.923 | 0.667 | 13,328 |
| 1.5 | 30 | 0.498 | 0.925 | 0.648 | 14,012 |
| 2.0 | 38 | 0.403 | 0.949 | 0.565 | 17,781 |
| 2.5 | 43 | 0.277 | 0.994 | 0.433 | 27,056 |
| 3.0 | 52 | 0.259 | 0.999 | 0.412 | 29,063 |

**Supplementary Table 3** Detailed information on the datasets used in this study.

| Dataset | Cells | Genes | Types | Accession | Description |
| --- | --- | --- | --- | --- | --- |
| 10X PBMC | 4271 | 16653 | 8 | Github (ttgump/scDeepCluster) | peripheral blood mononuclear cells |
| Airway | 7193 | 27716 | 7 | GSE103354 | mouse tracheal epithelium cells |
| Arc-ME | 20921 | 19743 | 36 | GSE93374 | mouse hypothalamic arcuate–median eminence complex |
| Colliculus | 26186 | 17482 | 28 | GSE223155 | mouse superior colliculus |
| Deng | 259 | 22431 | 10 | GSE45719 | mouse cells from different stages |
| Intestine | 6644 | 16328 | N/A | GSE123516 | irradiated mouse intestine crypts |
| Kidney_ccRCC | 20748 | 33694 | 13 | GSE159115 | Human kidney clear cell RCC |
| Kidney_normal | 6146 | 33694 | 26 | GSE159115 | Human kidney benign adjacent |
| Li | 561 | 57241 | 7 | GSE81861 | human colorectal tumors |
| Livers | 16015 | 19907 | 11 | GSE148339 | mouse frozen liver |
| MacParland | 8444 | 5000 | 11 | GSE115469 | human liver cells |
| Mammary | 13684 | 27998 | 13 | GSE150580 | murine mammary tissues |
| Pancreas | 8569 | 20125 | 14 | GSE84133 | human pancreas |
| Puram | 3363 | 23686 | 8 | GSE103322 | non-malignant cells in Head and Neck Cancer |
| Tcells | 73259 | 33538 | 13 | GSE164378 | T cells from human peripheral blood mononuclear |
| Tonsil | 5778 | 36601 | 13 | Broad Institute Single Cell Portal | human tonsil |
| UUOkidney | 6147 | 21516 | 17 | GSE119531 | mouse kidney from unilateral ureteral obstruction (UUO) |
| Embryos | 122278 | 52636 | N/A | GSE166692 | E14 mouse embryos sci-Space dataset |
| PBMC | 30669 | 14087 | 21 | GSE194122 | PBMC |

**Supplementary Table 4** The table displays the Jaccard similarity coefficients between scFormer-identified clusters and the original cell type annotations.

| scFormer Cluster | Basal | Ciliated | Club | Goblet | Lonocyte | Neuroendocrine | Tuft |
| --- | --- | --- | --- | --- | --- | --- | --- |
| 0 | 0 | 0.006494 | 0.263158 | 0.133333 | 0 | 0 | 0 |
| 1 | 0.255319 | 0 | 0 | 0 | 0 | 0 | 0.007353 |
| 2 | 0.204082 | 0 | 0 | 0 | 0 | 0.011236 | 0.007353 |
| 3 | 0.255319 | 0 | 0 | 0 | 0 | 0 | 0.007353 |
| 4 | 0 | 0.013072 | 0.315068 | 0.085106 | 0 | 0 | 0 |
| 5 | 0.082569 | 0 | 0 | 0.009901 | 0 | 0 | 0 |
| 6 | 0.017241 | 0.015284 | 0.185185 | 0.030303 | 0 | 0.011236 | 0.007353 |
| 7 | 0 | 0.006494 | 0.28 | 0.108696 | 0.026316 | 0 | 0 |
| 8 | 0.102804 | 0 | 0 | 0 | 0 | 0 | 0 |
| 9 | 0.255319 | 0 | 0 | 0 | 0 | 0 | 0 |
| 10 | 0 | 0.120482 | 0 | 0 | 0 | 0 | 0 |
| 11 | 0.008547 | 0.006494 | 0 | 0 | 0 | 0 | 0.007353 |
| 12 | 0 | 0.00432 | 0 | 0 | 0 | 0 | 0.37 |
| 13 | 0 | 0.006494 | 0 | 0 | 0.012987 | 0.267606 | 0.030075 |
| 14 | 0 | 0.00432 | 0.28 | 0.073684 | 0 | 0 | 0 |
| 15 | 0 | 0 | 0 | 0.478261 | 0.012987 | 0 | 0.007353 |
| 16 | 0 | 0 | 0 | 0 | 0.323529 | 0 | 0 |

**Supplementary Table 5** The table displays the Jaccard similarity coefficients between scFormer-identified clusters and the original cell type annotations.

| scFormer cluster | Original cluster | Cell type | Jaccard score |
| --- | --- | --- | --- |
| 0 | Cluster#17 | CBC | 0.346153846 |
| 1 | Cluster#4 | Enterocytes | 0.228070175 |
| 2 | Cluster#3 | Enterocytes | 0.352941176 |
| 3 | Cluster#1 | Enterocytes | 0.4 |
| 4 | Cluster#13 | Lymphocyte | 0.37254902 |
| 5 | Cluster#11 | Goblet | 0.4 |
| 6 | Cluster#13 | Lymphocyte | 0.129032258 |
| 7 | Cluster#15 | Paneth | 0.540540541 |
| 8 | Cluster#18 | SCC2 | 0.296296296 |
| 9 | Cluster#4 | Enterocytes | 0.186440678 |
| 10 | Cluster#12 | Goblet | 0.37254902 |
| 11 | Cluster#15 | Paneth | 0.540540541 |
| 12 | Cluster#10 | Enteroendocrine | 0.320754717 |
| 13 | Cluster#2 | Enterocytes | 0.4 |
| 14 | Cluster#13 | Lymphocyte | 0.272727273 |
| 15 | Cluster#5 | Enterocytes | 0.346153846 |
| 16 | Cluster#19 | Tuft | 0.4 |
| 17 | Cluster#14 | Lymphocyte | 0.044776119 |
| 18 | Cluster#14 | Lymphocyte | 0.014492754 |
